# Three pairs of surrogate redox partners comparison for Class I cytochrome P450 enzyme activity reconstitution

**DOI:** 10.1038/s42003-022-03764-4

**Published:** 2022-08-06

**Authors:** Xiaohui Liu, Fengwei Li, Tianjian Sun, Jiawei Guo, Xingwang Zhang, Xianliang Zheng, Lei Du, Wei Zhang, Li Ma, Shengying Li

**Affiliations:** 1 State Key Laboratory of Microbial Technology, Shandong University, Qingdao, Shandong 266237 China; 2 Laboratory for Marine Biology and Biotechnology, Qingdao National Laboratory for Marine Science and Technology, Qingdao, Shandong 266237 China; 3 Center For Biocatalysis and Enzyme Technology, AngelYeast Co., Ltd., Cheng Dong Avenue, Yichang, Hubei 443003 China

**Keywords:** Oxidoreductases, Metalloproteins

## Abstract

Most P450s require redox partners for the electron transfer during catalysis. However, little information is available on cognate redox partners for P450s, which greatly limits P450 function exploration and practical application. Thus, the stategy of building various hybrid P450 catalytic systems with surrogate redox partner has often adopted to engineer P450 biocatalysts. In this study, we compare three pairs of frequently-used surrogate redox partner *Sel*Fdx1499/*Sel*FdR0978, Adx/AdR and Pdx/PdR and in terms of their electron transfer properties. The three selected bacterial Class I P450s include PikC, P450sca-2 and CYP-sb21, which are responsible for production of high-value-added products. Here we show that *Sel*Fdx1499/*Sel*FdR0978 is the most promising redox partner compared to Adx/AdR and Pdx/PdR. The results provide insights into the domination for P450-redox partner interactions in modulating the catalytic activity of P450s. This study not only produces a more active biocatalyst but also suggests a general chose for a universal reductase which would facilitate engineering of P450 catalyst.

## Abstract (web summary)

Aiming for an efficient Class I cytochrome P450 catalytic system, three pairs of surrogate redox partners for biocatalyst applications are tested.

## Introduction

Cytochrome P450 enzymes (P450s or CYPs) are a superfamily of heme-thiolate proteins widespread in all kingdoms of life^1^. As one of the most versatile biocatalysts in nature^1,2^, P450s play crucial roles in natural product biosynthesis, biodegradation of xenobiotics, and bioproduction of pharmaceuticals and chemicals by catalyzing a myriad of oxidation reactions such as hydroxylation, epoxidation, decarboxylation, dealkylation, nitration, C–S bond formation, C–C bond cleavage, and aromatic coupling^3^.

Despite remarkable sequence diversity, most of P450s share a common three-demensional structural fold and catalytic cycle. The P450 catalysis is a complex cascade involving the protein-protein interactions between P450 and redox partners (RPs) as well as consumption of reducing equivalents^4^. There are two major P450 classes in terms of the native RP systems, namely, the prokaryotic Class I P450 consisting of three stand-alone components (redoxin reductase/redoxin/P450) that are all cytosolically soluble proteins and the two-component eukaryotic Class II P450 comprised of P450 and cytochrome P450 reductase (CPR), both of which are membrane-bound proteins. In the Class I apparatus, two electrons from NAD(P)H are transferred to the FAD-containing ferredoxin reductase (FdR) and then to P450 via the iron–sulfur cluster-containing ferredoxin (Fdx). This kind of RP systems are widely found in bacteria and the mitochondria in plants and animals, which serve many different physiological processes^5^.

Compared to the membrane-bound eukaryotic P450s, bacterial P450s in soluble form have better application value and potential. Although numerous P450 genes have been annotated, their innate RPs are normally difficult to identify due to their biological variations and/or difficulty to express^6^. Of note, the choice of RPs not only affect the substrate conversion rate but also the type and selectivity of reactions catalyzed by P450s^7^. Thus, a universal and efficient surrogate RP system is demanded for reconstituting the activities of bacterial P450s.

During the past years, a growing number of surrogate RPs have been reported^8^, with some of which being more frequently used for in vitro activity reconstitution of diverse bacterial P450s. For example, putidaredoxin (Pdx) and putidaredoxin reductase (PdR) from *Pseudomonas putida* represent the first discovered bacterial P450 RP system served as the native PR for P450cam (CYP101A1) in camphor oxidation^9,10^. Bovine adrenodoxin (Adx) and adrenodoxin reductase (AdR) are involved in steroid hormone biosynthesis in the adrenal gland mitochondria. Although Adx/AdR are originated from a eukaryotic source, they are able to support many prokaryotic P450s such as the steroid hydroxylase CYP106A2 from *Bacillus megaterium*^11^ and CYP109D5 from *Sorangium cellulosum* So ce56^12^. Recently, this and other laboratories revealed that the Fe_2_S_2_ ferredoxin *Sel*Fdx1499 and plastidic-type ferredoxin reductase *Sel*FdR0978 from *Synechococcus elongatus* PCC 7942 showed high and broad supporting activities for a growing number of bacterial Class I P450s, such as MycG (CYP107E1) from *Micromonospora griseorubida*, PikC (CYP107L1) from *Streptomyces venezuelae*, CreJ (CYP288A2) from *Corynebacterium glutamicum*, CYP105AS-1 from *Amycolatopsis orientalis*, and P450sca-2 (CYP105A3) from *Streptomyces carbophilus*^13–16^.

Interestingly, the Fe_2_S_2_ Fdxs have been found to be more likely to provide high electron transfer efficiency for bacterial Class I P450s^13^. Aiming to find out the best RP pairs among the above-mentioned popular surrogate RPs, in this work, we constituted a small reaction matrix with the three Fe_2_S_2_ Fdxs and their corresponding FdRs (i.e., *Sel*Fdx1499/*Sel*FdR0978, Adx/AdR, and Pdx/PdR) to compare their electron transfer efficiencies, P450-supporting activities, and recognition modes between different RPs and P450s. Of note, a more stable and active mutant Adx(4–108) instead of the full-length Adx was used in this study and this truncated mutant will be presented by Adx for convenience. The three chosen Class I P450 enzymes were PikC (CYP107L1) from *S. venezuelae*, P450sca-2 (CYP105A3) from *S. carbophilus*, and CYP-sb21 (CYP107Z14) from *Sebekia benihana*. Further structural analysis and molecular docking studies led to more understandings of the protein-protein interactions in the P450-Fdx and Fdx-FdR complexes, which are critical for shuttling of reducing equivalents and affect P450 activity. We expect that the key findings in this work will provide more mechanistic and practical guidance for construction of an efficient Class I P450 catalytic system.

## Results

### Spectral properties of the purified P450s and surrogate RPs

The three selected P450s and three pairs of surrogate RPs were successfully expressed and purified. All these proteins were purified to homogeneity as shown by SDS-PAGE analysis (Supplementary Fig. 1). The CO-bound reduced difference spectra of PikC, P450sca-2, and CYP-sb21 showed a characteristic peak at 450 nm, indicative of functional P450 enzymes (Supplementary Fig. 2). The absorption spectra of the three flavoproteins (*Sel*FdR0978, AdR, and PdR) gave the signature peaks at around 410 nm, 455 nm, and a shoulder at 395 nm. The three Fe_2_S_2_ cluster-containing proteins (*Sel*Fdx1499, Adx, and Pdx) displayed the typical UV-Vis spectra with two local maximal peaks at approximately 380–420 nm and 460 nm (Supplementary Fig. 3). The extinction coefficients of the flavin-bound *Sel*FdR0978 and the iron–sulfur containing *Sel*Fdx1499 were determined to be *ε*_454_ = 19,120 M^−1^ cm^−1^ and *ε*_460_ = 11,270 M^−1^ cm^−1^, respectively.

### The P450 activities supported by different surrogate RPs

**Fig. 1:**
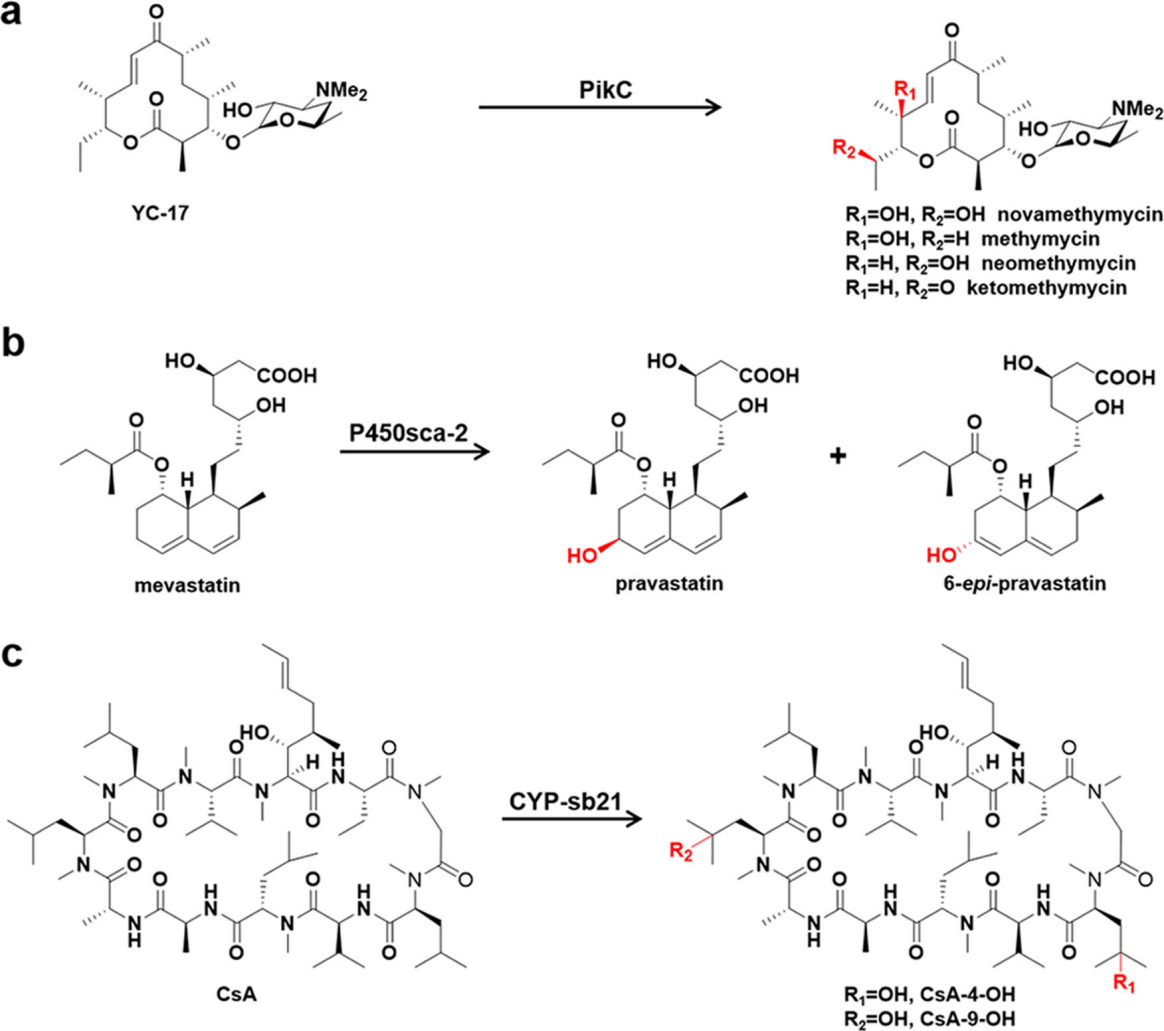
The P450-catalyzed reactions. **a** PikC hydroxylates YC-17 to produce the mono-hydroxylated products methymycin and neomethymycin, and two dioxygenation products novamethymycin and ketomethymycin. **b** P450sca-2 hydroxylates mevastatin to form pravastatin and 6-*epi*-pravastatin. **c** CYP-sb21 catalyzes the oxidation of cyclosporine A (CsA) to *γ*-hydroxy-*N*-methyl-L-Leu4-CsA (CsA-4-OH) and *γ*-hydroxy-*N*-methyl-L-Leu9-CsA (CsA-9-OH). Functional groups and bonds colored in red are catalyzed by the P450s.

The choice of surrogate RPs (when the innate RPs are unavailable) is crucial for activity reconstitution of P450s. To determine the optimal surrogate RPs for Class I P450 enzymes, a small reaction matrix with three surrogate RP systems and three P450s (against 3 substrates) was constituted. Regarding the P450 catalytic activities, PikC is able to hydroxylate the 12-membered ring macrolactone YC-17 to produce the mono-hydroxylated products methymycin and neomethymycin, and two dioxygenation products novamethymycin and ketomethymycin (Fig. 1a)^17^. P450sca-2 is an industrially important enzyme that can hydroxylate mevastatin to pravastatin as the desired product and the side product 6-*epi*-pravastatin (Fig. 1b)^18,19^. CYP-sb21 is capable of catalyzing the site-specific hydroxylation of the immunosuppressant cyclosporine A (CsA), giving rise to the valuable hair-growth-stimulating agent *γ*-hydroxy-*N*-methyl-L-Leu4-CsA (CsA-4-OH)^15^ (Fig. 1c).

**Fig. 2:**
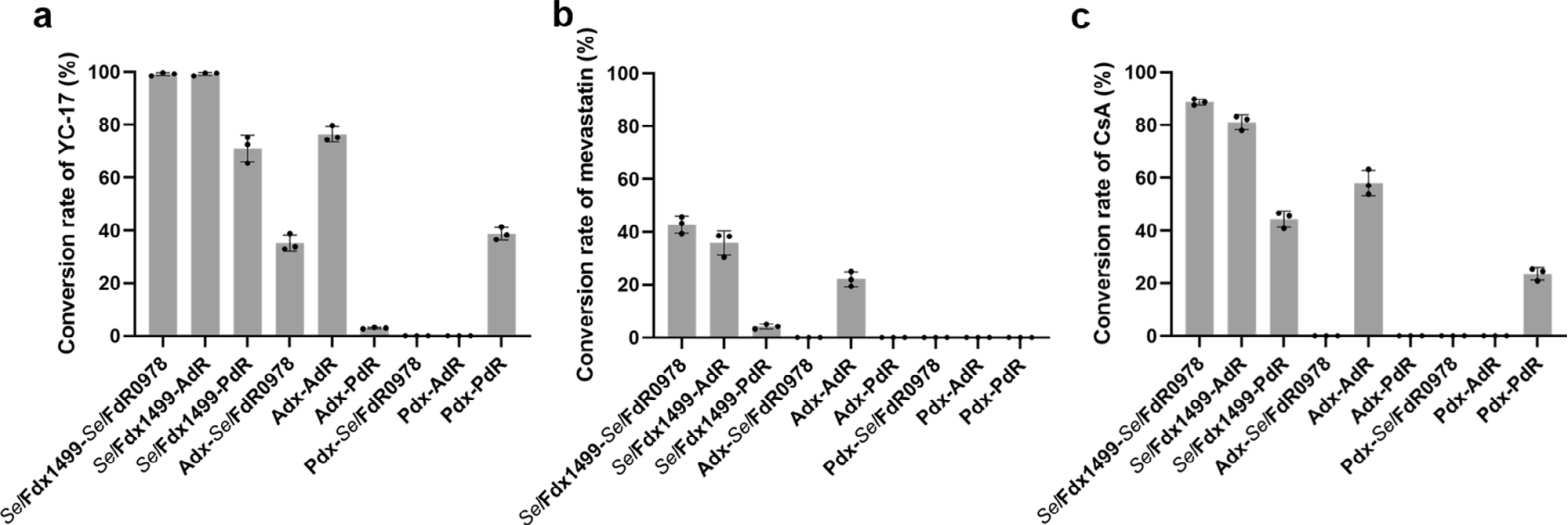
Influence of the selection of redox partner on P450 activities. The catalytic activities of PikC (**a**), P450sca-2 (**b**), and CYP-sb21 (**c**) when supported by nine different combinations of redoxin and redoxin reductase. All the experiments were carried out in triplicate. The error line represents the standard deviation. *P* values in each groups were calculated with single factor ANOVA analysis, and all *P* values were <0.01.

In the reaction matrix, the 27 P450 reactions resulted from three P450s and nine (3 × 3) different RP combinations of *Sel*Fdx1499/Adx/Pdx and *Sel*FdR0978/AdR/PdR were analyzed by HPLC. Interestingly, the product profiles varied with the choice of RPs (Fig. 2 and Supplementary Fig. 5). When partnering with PikC, nine pairs of RPs resulted in a broad range of substrate conversion rates and different product distributions. Specifically, *Sel*Fdx1499/*Sel*FdR0978, *Sel*Fdx1499/AdR, and *Sel*Fdx1499/PdR led to 99.1%, 99.3%, and 71.1% conversions of YC-17 by PikC, while the corresponding RP pairs with *Sel*Fdx1499 replaced by Adx gave 35.2%, 76.4%, and 3.0% conversions, respectively. For Pdx, the only active pair was Pdx/PdR (Fig. 2a). With regard to product distributions, the three *Sel*Fdx1499 combinations (*Sel*Fdx1499/*Sel*FdR0978, *Sel*Fdx1499/PdR, *Sel*Fdx1499/AdR) and the Adx/AdR produced the dioxygenation products of YC-17, the ratios of four procucts (methymycin:neomethymycin:novamethymycin:ketomethymycin) were 30.5:43.5:20.6:5.4, 9.1:49.7:34.5:6.7, 2.1:52.8:43.4: .7, and 4.1:51.3:43.5:1, respectively (Supplementary Figs. 5a, 6, and 7). These results suggested that *Sel*Fdx1499/*Sel*FdR0978 or *Sel*Fdx1499/AdR could provide higher electron transfer efficiency, thus generating more dihydroxylated products.

P450sca-2 produced pravastatin as the main product in all positive reactions, and only negligible amounts of 6-*epi*-pravastatin as detected by high-resolution mass spectrometry (HRMS) (Fig. 2b, Supplementary Figs. 5b, and 7). The combination of *Sel*Fdx1499 with *Sel*FdR0978, AdR, or PdR resulted in 43.0%, 35.5%, and 7.3% of substrate consumption, respectively. When Adx was coupled with AdR, the conversion rate of mevastatin by P450sca-2 reached 22.1%. Pdx coupled with *Sel*FdR0978 and PdR showed low activity for P450sca-2, and only negligible amounts of pravastatin as detected by HRMS. However, no product was detected when Pdx coupled with AdR (Fig. 2b and Supplementary Fig. 5b).

For CYP-sb21, >80% conversions of the substrate CsA were observed when *Sel*Fdx1499 was coupled with *Sel*FdR0978 (86.7%) or AdR (81.1%), and the ratios of the two products CsA-4-OH and CsA-9-OH were 9.2:1 and 3.9:1, respectively (Fig. 2c, Supplementary Figs. 5c and 7). Adx/AdR or Pdx/PdR were able to support the hydroxylating activity of CYP-sb21 with the ratios of 57.9% and 23.6%, respectively. However, no product was detected when Adx or Pdx was combined with non-cognate redoxin reductases (Fig. 2c and Supplementary Fig. 5c), indicating that Adx/AdR and Pdx/PdR are more strict RP pairs than *Sel*Fdx1499/*Sel*FdR0978.

It is worth mentioning that we investigated the effect of P450/Fdx/FdR ratio on P450-mediated catalysis before the above-described activity comparisons. The effects of different P450/Fdx/FdR ratios were well studied by this and other laboratories and the ratio of 1:10:5 was often used^13,20,21^. Importantly, even in the case of natural P450 redox chain of PdR/Pdx/P450cam, an excess of Pdx was reported to be required for efficient catalysis^22^. To optimize the P450 activities, in this study, we re-examined the P450/Fdx/FdR ratios of 1:1:1, 1:10:1, 1:10:5, and 1:20:10. In general, the ratio of 1:10:5 led to a higher substrate conversion than that of 1:1:1 (Supplementary Fig. 4). For the hybrid *Sel*Fdx1499/PdR system, the ratios of 1:1:1, 1:10:1, 1:10:5, and 1:20:10 gave 0%, 3.4%, 78.3%, and 88.5% conversions of YC-17 by PikC, respectively. Among the tested ratios, 1:10:5 and 1:20:10 exhibited relatively higher activities. Although the 1:20:10 system gave a slightly higher conversion rate, considering the saving of materials and the fact that a high FdR concentration could lead to increased nonspecific oxidation of NAD(P)H^23^, the ratio of 1:10:5 was chosen to drive the P450-mediated reactions.

We also determined the NAD(P)H coupling efficiencies of the 27 P450 reactions (Supplementary Table 1) by following the previously established procedure^24^. The three cognate combinations (*Sel*Fdx1499/*Sel*FdR0978, Adx/AdR, and Pdx/PdR) exhibited higher coupling efficiencies than the corresponding hybrid RP combinations. *Sel*Fdx1499/*Sel*FdR0978 demonstrated the highest coupling efficiency in all three P450 reaction systems. The CYP-sb21 system only achieved the highest coupling efficiency of 12.6% when partnered by *Sel*Fdx1499/*Sel*FdR0978, which is in good agreement with the low activity towards CsA^15^. The low NAD(P)H coupling efficiency is an important limiting factor for the activity of a reconstituted P450 system^15^. Thus, an NADPH regeneration system (GDH/glucose) was used in this study to increase the P450 activities.

### The reductase activities and cofactor preference of FdRs

**Fig. 3:**
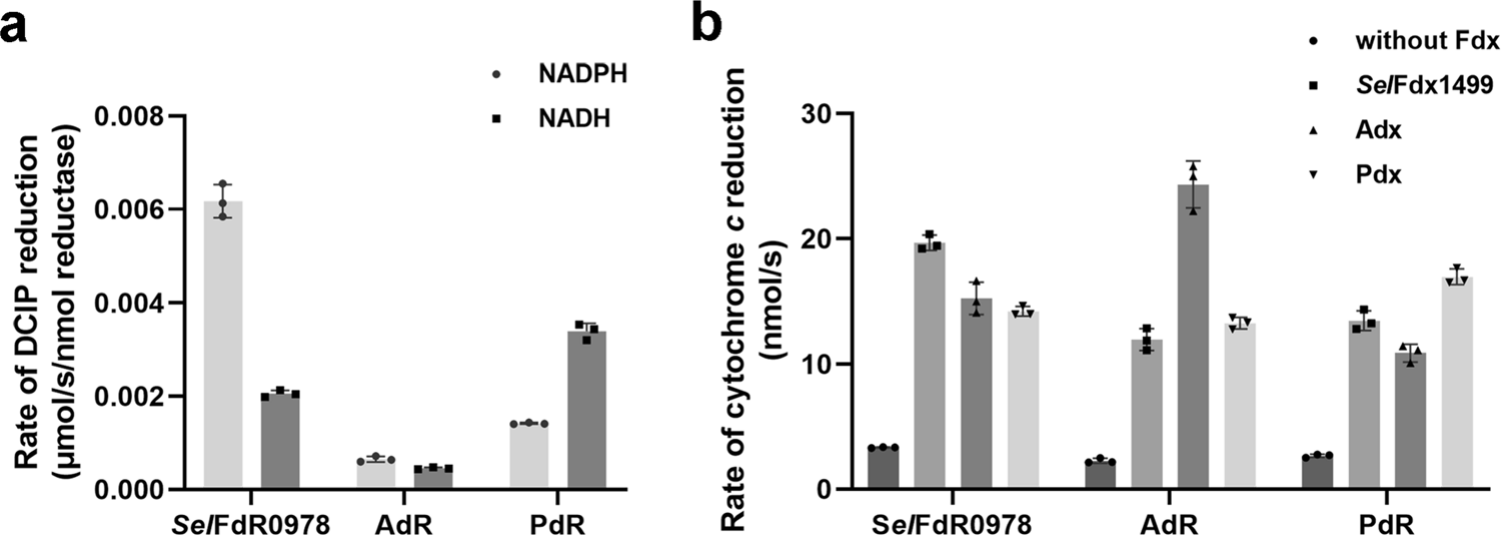
DCIP and cyt c reduction assays. **a** DCIP reduction activities of the three redoxin reductases in the presence of 1 mM NADH or NADPH. **b** Rates of electron transfer measured by cyt *c* reduction. The reaction mixture contained 30 μM cyt *c*, 10 nM FdR or 10 nM FdR coupled with 20 nM Fdx, and started by adding 500 μM NAD(P)H. All experiments were independently repeated for three times. The error line represents the standard deviation. *P* values in each groups were calculated with single factor ANOVA analysis, and all *P* values were <0.01.

The reductase activities of FdRs were determined using 2,6-dichloroindophenol (DCIP) as an electron acceptor. As a result, *Sel*FdR0978 was more efficient in DCIP reduction than both AdR and PdR. Electron donor preference was investigated by changing NADPH to NADH in the reaction mixtures. All the three FdRs were able to use either NADPH or NADH as electron donor. *Sel*FdR0978 showed a strong preference for NADPH over NADH (3-fold increase), while AdR only exhibited a slightly increased activity with NADPH than with NADH. As previously reported^25^, PdR preferred NADH to NADPH. When the preferred electron donor was used for the three FdRs, *Sel*FdR0978 gave the highest DCIP reduction, which was approximately 2 and 9 times higher than PdR and AdR, respectively (Fig. 3a).

The electron transfer efficiency of different RP systems (NAD(P)H → redoxin reductase → redoxin → cytochrome *c*). Cytochrome *c* (cyt *c*) is routinely used as an electron acceptor to evaluate the electron transfer efficiency of different RPs^26,27^. The constructed nine (3 × 3) different RP combinations showed a broad range of NAD(P)H-dependent cyt *c* reductase activities (Table 1). Consistent with the observations made in previous studies^26,28,29^, the three FdRs were found to directly transfer electrons to cyt *c* more slowly in the absence of Fdx (Fig. 3b), thus having minor effects on cyt *c* reduction by Fdx. Among the tested three redoxins, as expected, the combinations with their cognate redoxin reductases gave a higher activity of cyt *c* reduction. *Sel*Fdx1499 was determined to be the most efficient redoxin with the prominent high *k*_cat_/*K*_m_ values when coupled with *Sel*FdR0978 (*k*_cat_/*K*_m_ = 0.92 μM^−1^ s^−1^) or AdR (*k*_cat_/*K*_m_ = 0.85 μM^−1^ s^−1^). Both combinations demonstrated about 4-fold higher *k*_cat_/*K*_m_ values when compared with that of *Sel*Fdx1499/PdR (*k*_cat_/*K*_m_ = 0.23 μM^−1^ s^−1^). Adx was also an efficient redoxin when paired with AdR (*k*_cat_/*K*_m_ = 0.27 μM^−1^ s^−1^) and *Sel*FdR0978 (*k*_cat_/*K*_m_ = 0.16 μM^−1^ s^−1^). Adx and Pdx showed a minor difference when combined with *Sel*FdR0978. The combinations of Adx/PdR and Pdx/AdR showed the lowest reducing efficiencies with *k*_cat_/*K*_m_ values of 0.004 and 0.09 μM^−1^ s^−1^, respectively (Table 1 and Supplementary Fig. 8). An overview of the *K*_m_ values suggested that all three redoxins could form an efficient electron transfer chain with their corresponding cognate FdRs. *Sel*Fdx1499/AdR gave the highest *k*_cat_ value (89.5 ± 25.5 s^−1^) among the tested RP pairs. All other RP pairs including the three cognate pairs showed >79% lower *k*_cat_ values relative to that of *Sel*Fdx1499/AdR (Table 1). In general, the presence of *Sel*Fdx1499 or *Sel*FdR0978 led to a relatively higher *k*_cat_ value for unknown reasons.

**Table 1:** Kinetic parameters for reduction of redoxins (*Sel*Fdx1499, Adx, Pdx) by redoxin reductases (*Sel*FdR0978, AdR, PdR) using cyt *c* as the electron acceptor^a^.

Electron transfer chain	*K*_m_ (μM)	*k*_cat_ (s^−1^)	*k*_cat_/*K*_m_ (μM^−1^ s^−1^)
*Sel*Fdx1499/*Sel*FdR0978	14.2 ± 1.7	13.1 ± 0.8	0.92
*Sel*Fdx1499/AdR	105.1 ± 36.3	89.5 ± 25.5	0.85
*Sel*Fdx1499/PdR	62.1 ± 13.9	14.5 ± 2.2	0.23
Adx/*Sel*FdR0978	27.8 ± 9.0	4.7 ± 0.8	0.16
Adx/AdR	14.6 ± 2.7	4.0 ± 0.3	0.27
Adx/PdR	57.9 ± 25.1	0.2 ± 0.1	0.004
Pdx/*Sel*FdR0978	64.2 ± 8.6	18.4 ± 1.7	0.28
Pdx/AdR	48.4 ± 16.2	4.5 ± 0.9	0.09
Pdx/PdR	22.1 ± 4.8	10.5 ± 1.1	0.47

^a^All experiments were performed in triplicate, and all standard errors were <10%.

### Electrostatic surface analysis and interprotein docking of P450s and RPs

**Fig. 4:**
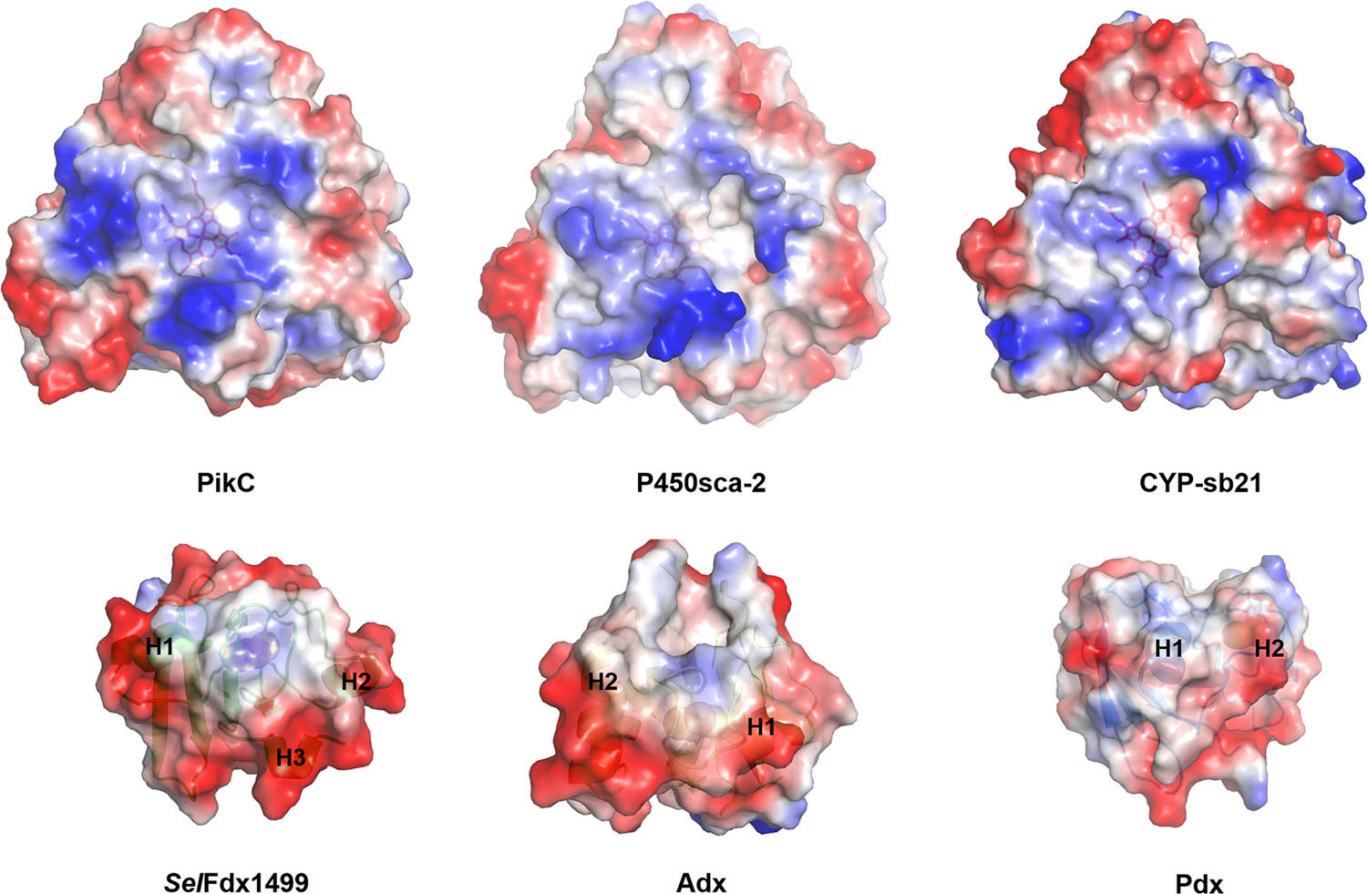
Electrostatic surface analysis of P450s and Fdxs. Positively and negatively charged surfaces are colored in blue and red, respectively. The electrostatic surfaces of Fdxs with Helix1 (H1), Helix2 (H2), or Helix3 (H3) are marked. The six proteins are PikC/YC-17 (PDB ID: 2VZ7), P450sca-2 (predicted by Alphafold2), CYP-sb21 (substrate-free, PDB ID: 6M4S), *Sel*Fdx1499 (predicted by Alphafold2), Adx (PDB ID: 1CJZ), and Pdx (PDB ID: 1PDX).

**Fig. 5:**
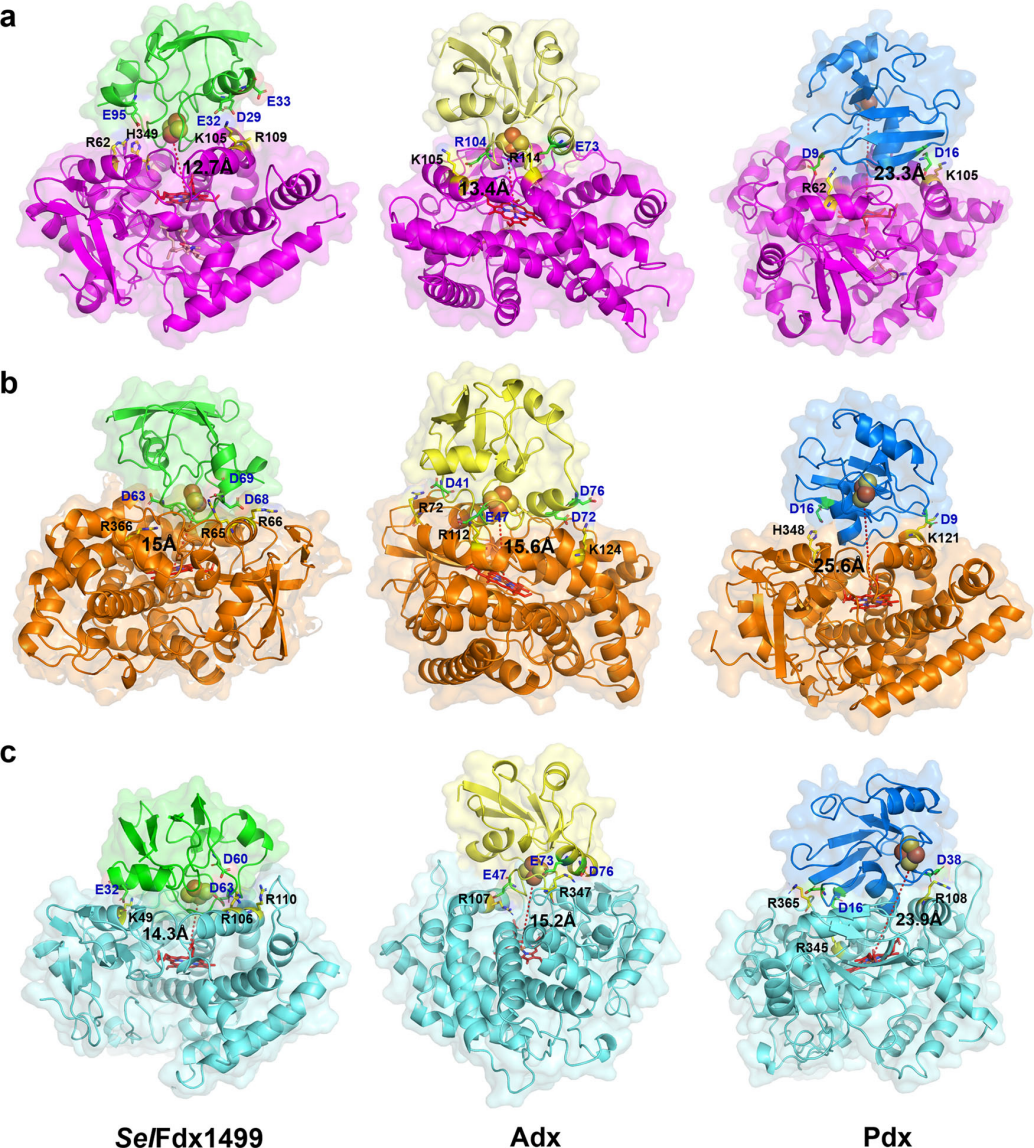
Fdx-P450 docking models. Docking models of three Fdxs in complex with PikC (**a**), P450sca-2 (**b**), and CYP-sb21 (**c**). The structures of P450-Fdx complexes are shown as cartoon with different colors. The key interacting residues on P450-Fdx interfaces are shown as sticks in yellow and green, respectively. Heme and substrates are shown as sticks in red and wheat, respectively. The Fe_2_S_2_ cluster are shown as spheres. The distances (Å) between the iron–sulfur cluster and heme-iron are indicated by dashed red lines.

Alphafold2 was used to predict the unsolved crystal structures of P450sca-2, *Sel*FdR0978, and *Sel*Fdx1499^30^. Then, the computer models for the P450-Fdx and FdR-Fdx ET complexes were generated by ZDOCK Server (ZDOCK 3.0.2)^31^. As expected, the three Fdxs unanimously bind onto the proximal side of P450s. To understand the recognition mechanism of the nine P450-Fdx pairs, the electrostatic surfaces were analyzed. PikC, P450sca-2, and CYP-sb21 have some conserved Lys, Arg and His residues that form a positively charged feature on their proximal surfaces (Supplementary Figs. 9 and 10). By contrast, Fdxs harbor a number of acidic amino acids surrounding the hydrophobic Fe_2_S_2_ cluster (Fig. 4). The acidic and basic amino acids shown on the protein-protein interfaces were predicted to be involved in formation and stabilization of these P450-Fdx complexes (Fig. 5). Notably, because the three Fdxs have different electrostatic and interaction surfaces, they interact with the same P450 via different acidic amino acids, thus adopting different interacting orientations. For example, in the CYP-sb21-*Sel*Fdx1499 complex, E32 (Helix1 of Fdx) interacts with K49 (Helix B of CYP-sb21), while D60 and D63 interact with R106 and R110 (Helix C of CYP-sb21) through electrostatic contacts. In the CYP-sb21-Adx complex, E47 interacts with K107 (Helix C of CYP-sb21), while E73 and D76 (Helix3 of Adx) electrostatically interact with R347. *Sel*Fdx1499 and Adx demonstrate a more negatively charged surface than Pdx, which might have greater opportunities to form an ideal interface with various P450 enzymes, thereby achieving more efficient electronic transmission. For the three *Sel*Fdx1499-P450 complexes displaying high substrate conversions, the distances between the iron–sulfur cluster and heme-iron in the most likely docking modes range from 12.7 to 15 Å, which are shorter than the corresponding distances observed in the Adx-P450 and Pdx-P450 complexes. The results indicated that a shorter Fe_2_S_2_-to-heme-iron distance would likely increase the electron transfer efficiency. Furthermore, *Sel*Fdx1499 has a larger anionic convex surface than both Adx and Pdx (Figs. 4 and 5). Taken together, the intensity of negatively charged surface, the charge distribution of Fdxs, and the distance between iron–sulfur cluster and heme-iron appear to be the key factors to determine the electron transfer efficiency between redoxin and P450.

**Fig. 6:**
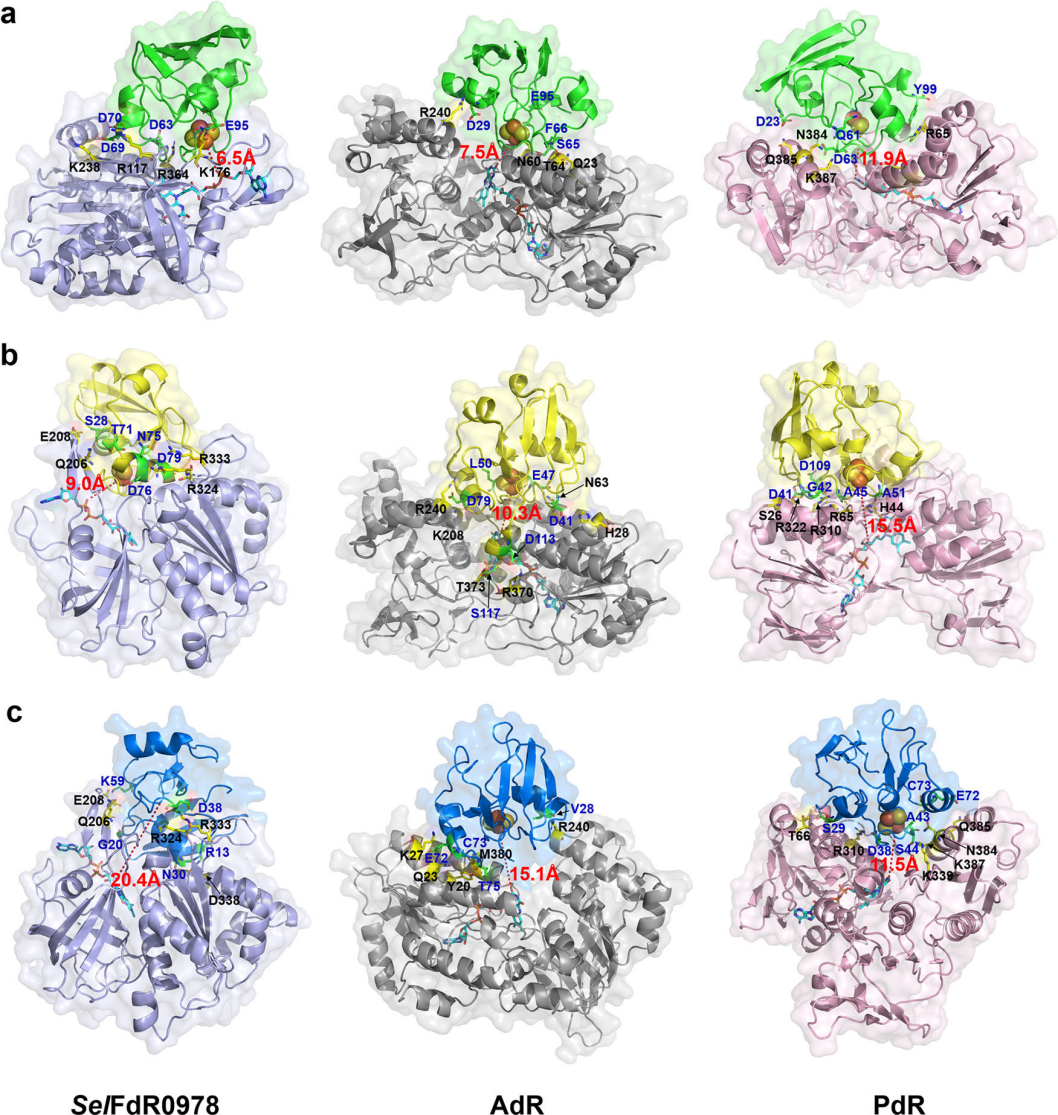
Fdx-FdR docking models. Docking models of three FdRs in complex with *Sel*Fdx1499 (**a**), Adx (**b**), and Pdx (**c**). The structures of FdR-Fdx complexes are shown as cartoon with different colors. The key interacting residues on FdR-Fdx interfaces are shown as sticks in yellow and green, respectively. FAD is shown as sticks in cyan. The Fe_2_S_2_ cluster is shown as spheres. The shortest FAD-Fe_2_S_2_ distances (Å) are indicated by dashed red lines.

The electron transfer efficiency between redoxin reductase and redoxin could also influence the overall electron transfer rate and the catalytic efficiency of P450s. Some progresses have been made in general understanding of the interactions of AdR-Adx as well as PdR-Pdx based on their crystal structures^32,33^. However, the crystal structure of *Sel*Fdx1499-*Sel*FdR0978 complex has not yet been successfully obtained. Thus, we built a model of *Sel*FdR0978-*Sel*Fdx1499 using ZDOCK software, in which the interface is ~522.8 Å^2^ of the surface area in each protein. For AdR-Adx and PdR-Pdx, the interface areas are 580 Å^2^ and 365 Å^2^, respectively^32,33^. In the X-ray structure of the covalent cross-linked AdR-Adx and PdR-Pdx complexes, the electrostatic forces were found to play an important role in formation and stabilization of productive ET complexes. Among the interface residues in PdR-Pdx, there are only one bidentate Arg^310^PdR-Asp^38^Pdx salt bridge and five hydrogen bonds^33^. The interface of AdR-Adx is composed of His^28^AdR-Asp^41^Adx salt bridge and several interprotein hydrogen bonds. In the ZDOCK model of *Sel*Fdx1499-*Sel*FdR0978, Asp^70^ and Asp^63^ of *Sel*Fdx1499 interact with Arg^117^ and Arg^364^ of *Sel*FdR0978 by forming a salt bridge, respectively. In combination with other interprotein hydrogen bonds, a stable conformation convenient for ET is formed. The shortest FAD-Fe_2_S_2_ distance for *Sel*FdR0978 with *Sel*Fdx1499, Adx, and Pdx were determined to be 6.5 Å, 9.0 Å, and 20.4 Å, respectively. For AdR (or PdR), the shortest FAD-Fe_2_S_2_ distance were found to be 7.5 Å (11.9 Å), 10.3 Å (15.5 Å), and 15.1 Å (11.5 Å), when coupled with *Sel*Fdx1499, Adx, and Pdx, respectively (Fig. 6). The docking results of the three pairs of RPs agree well with the experimental data and suggest that a proper protein-protein interaction (PPI) conformation would directly determine the distance between FAD and iron–sulfur cluster, which is one of the key factors affecting ET efficiency.

To validate the ZDOCK models, the amino acid residues on the predicted Fdx interaction interfaces were replaced by alanine in a grouped manner. The conversion of YC-17 by PikC was selected for activity evaluation of different Fdx mutants including *Sel*Fdx1499-M1−M4, Adx-M1−M4, and Pdx-M1−M4. As expected, a majority of reactions showed a decreased substrate conversion rate when partnered by different Fdx mutants (Table 2). It is intriguing that some Fdx mutants with their PPI interface charges removed gave an even higher YC-17 conversion rate than the corresponding wild-type Fdxs. For example, first, compared with the parental *Sel*Fdx1499, *Sel*Fdx1499-M2, and *Sel*Fdx1499-M3 led to an improved YC-17 conversion when partnered with AdR and PdR, respectively. Structure modeling showed that a negative interaction surface can still form (Supplementary Fig. 11a–c). Protein-protein docking analysis of *Sel*Fdx1499-M2/AdR and *Sel*Fdx1499-M3/PdR revealed that the mutations induce the change of their corresponding PPI interfaces with AdR and PdR. These alternative PPIs resulted in closer distances between the iron–sulfur cluster and heme-iron compared to those for wild-type *Sel*Fdx1499 (Supplementary Fig. 11d, e). Thus, we reason that Fdx may harbor redundant negatively charged residues on the surface to recognize and interact with different P450s and FdRs. This redundancy could explain the tolerance of the interface residues to multiple charge-removing mutations. Second, Adx-M4 with a changed Fdx-P450 PPI interface gave a higher PikC activity than the parental Adx (76.3% *versus* 63.8%). Third, the double mutations in Pdx-M4 even enabled the activity of the PikC/Pdx/*Sel*FdR0978 system. Taken together, these results strongly suggest that the prediction of PPI interface residues based on P450-Fdx and Fdx-FdR docking should be reliable and practical, and these amino acids are important for the efficient catalysis by P450s.

**Table 2:** Conversions of YC-17 by PikC when Fdx mutants coupled with different FdRs^a^.

Fdx	Mutation sites	Characteristics	Conversion rates of YC-17		
			Coupled with *Sel*FdR0978	Coupled with AdR	Coupled with PdR
*Sel*Fdx1499	–	Wild type	99.1 ± 0.1	30.1 ± 1.6	24.7 ± 0.9
*Sel*Fdx1499-M1	D63A/D69A/D70A/E95A	Multiple mutations of the residues involved in *Sel*Fdx1499-*Sel*FdR0978 interaction	86.6 ± 1.0	–	–
*Sel*Fdx1499-M2	D29A/S65A/F66A/E95A	Multiple mutations of the residues involved in *Sel*Fdx1499-AdR interaction	–	65.4 ± 0.9	–
*Sel*Fdx1499-M3	D23A/Q61A/D63A/Y99A	Multiple mutations of the residues involved in *Sel*Fdx1499-PdR interaction	–	–	40.3 ± 2.2
*Sel*Fdx1499-M4	D29A/E32A/E33A/E95A	Multiple mutations of the residues involved in *Sel*Fdx1499-PikC interaction	69.2 ± 1.2	19.3 ± 2.0	37.1 ± 1.5
Adx	–	Wild type	15.6 ± 1.7	63.8 ± 0.2	16.5 ± 0.5
Adx-M1	S28A/T71A/N75A/D76A/D79A	Multiple mutations of the residues involved in Adx-*Sel*FdR0978 interaction	9.2 ± 0.5	–	–
Adx-M2	D41A/E47A/L50A/D79A	Multiple mutations of the residues involved in Adx-AdR interaction	–	67.8 ± 1.1	–
Adx-M3	D41A/G42A/A45L	Multiple mutations of the residues involved in Adx-PdR interaction	–	–	7.2 ± 0.1
Adx-M4	E73A/R104A	Multiple mutations of the residues involved in Adx-PikC interaction	5.1 ± 0.8	76.3 ± 0.9	4.2 ± 0.1
Pdx	–	Wild type	0	0	15.6 ± 0.2
Pdx-M1	R13A/G20A/N30A/D38A/K59A	Multiple mutations of the residues involved in Pdx-*Sel*FdR0978 interaction	0	–	–
Pdx-M2	V28K/E72A/T75K	Multiple mutations of the residues involved in Pdx-AdR interaction	–	0	–
Pdx-M3	S29A/D38A/E72A	Multiple mutations of the residues involved in Pdx-PdR interaction	–	–	6.5 ± 0.6
Pdx-M4	D9A/D16A	Multiple mutations of the residues involved in Pdx-PikC interaction	11.7 ± 1.1	0	9.6 ± 0.9

^a^The standard assay contained 1 μM P450, 10 μM Fdx, 5 μM FdR, 200 μM substrate, 0.5 mM NAD(P)^+^, and 10 mM glucose plus 5 U glucose dehydrogenase as NAD(P)H regeneration system. All experiments were performed in triplicate.

## Discussion

The three-component Class I P450 system consists of an iron–sulfur-containing redoxin, an FAD-containing redoxin reductase, and a P450 monooxygenase. The catalytic rate of such a system is often limited by the rate of ET from RPs to P450^13^. The two-electron carrier FdR is responsible for shuttling two electrons from NAD(P)H to Fdx. The reduced one-electron carrier Fdx sequentially delivers two electrons to P450 to support the activation of dioxygen for substrate oxidation. A rapid ET from RP proteins to P450 requires effective PPIs of both FdR-Fdx and P450-Fdx, and appropriate reductive potential differences^6^.

The PPIs between FdR-Fdx-P450 during electron transport have not yet been fully understood primarily because of the lack of co-crystal structures for the two- or three-component complex. To date, only little structural information of RP couples is available, such as PdR-Pdx (PDB code 3LB8) and AdR-Adx (PDB code 1E6E)^32,33^. For these redox couples, the electrostatic/hydrophobic interactions, salt bridge-forming residues, and peripheral hydrogen bonds play essential roles in formation and stabilization of productive ET complexes^34^. The AdR-Adx complex is remarkably similar to PdR-Pdx in terms of binding topology and geometry^32^. Although the sequence identities for Adx/Pdx and AdR/PdR are only 32.4% and 19.4% (Supplementary Fig. 10), respectively, Pdx and Adx share a highly conserved ^86^SRLXCQ^91^ motif. Interestingly, His54, which was identified to play a significant role for AdR recognition, is absolutely conserved in vertebrate-type and some bacterial-type ferredoxins (e.g., Pdx)^9^, but not found in *Sel*Fdx1499 (Supplementary Fig. 10). Although Adx and Pdx have some similar features when compared with *Sel*Fdx1499, the activities of three P450s were low when paired with AdR/Pdx or PdR/Adx. This suggests that except the key residues and common manner of RPs interactions, the interface residues and proper distance between FdR and Fdx are also important factors. Cognate RPs including *Sel*Fdx1499/*Sel*FdR0978, Adx/AdR, and Pdx/PdR were active in all the three tested P450 reactions, indicating that all the three redoxins can deliver electrons to the selected P450s. Among the 27 reactions with different Fdx/FdR combinations, 74.1% of combinations successfully supported the P450 activities. Besides the desired interaction between iron–sulfur cluster and P450, it is also worth noting that the recognition between RPs could affect the Fdx-to-P450 electron transfer efficiency.

Fdx-P450 recognition is primarily dependent on the electrostatic interaction and especially important in the catalytic cycle of P450. It was thought that proper Fdx binding would result in changes in the spectral and physical properties of P450^35^. In the well-studied P450cam catalytic system, the salt bridge between Pdx Asp^38^ and P450cam Arg^112^ plays a vital role in the binding and ET in the Pdx-P450cam complex^35^. The comparison reveals a number of Arg, His, and Lys residues that are conserved among these three P450 enzymes (Supplementary Figs. 9 and 10).

The effect of ionic strength on the interactions of P450/Fdx/FdR was investigated in a serial NaCl concentrations ranging from 100 to 1000 mM at a constant pH of 7.4. As expected, the conversion rates of YC-17 by PikC when coupled with all three cognate RP pairs gradually decreased along with the increasing NaCl concentrations (Supplementary Fig. 12). These results support that electrostatic interactions play an important role in electron transfer from RPs to P450.

In this study, we clearly showed that *Sel*Fdx1499/*Sel*FdR0978 is the most effective and efficient RPs to support the three P450 hydroxylases. An interesting question arose whether this finding is also true for other types of P450 enzymes. To address this issue, we further examined the supporting activities of surrogate RPs towards the Class I P450 epoxidase EpoK (CYP167A1). EpoK catalyzes the epoxidation of C12-C13 double bond in epothilone D to form epothilone B^36^ (Supplementary Fig. 13a). Among the nine combinations of surrogate RPs, the combinations of *Sel*Fdx1499/*Sel*FdR0978 and Adx/AdR were able to reconstitute the epoxidation activity of EpoK, with the substrate conversion rates of 97% and 6%, respectively (Supplementary Fig. 13). Of note, *Sel*Fdx1499/*Sel*FdR0978 exhibited a higher supporting activity for EpoK than the previously reported spinach RPs^37^. In conclusion, *Sel*Fdx1499/*Sel*FdR0978 seems to be an optimal choice for Class I P450 activity reconstitutions.

In a P450 catalytic cycle, RPs often limit the overall reaction rate. Due to the weak PPI and quick inactivation of Fdx, it is difficult to obtain the co-crystallized P450-RP and redox complexes. Therefore, the lack of structures for both redox complexes has hindered the understanding of the details during RP-P450 interactions. In this work, we investigated three typical prokaryotic RP pairs in order to find a convenient way to guide the selection of RPs for a specific Class I P450 reaction. Based on our results, we suggest that *Sel*Fdx1499/*Sel*FdR0978 should be utilized more generally in the future.

## Methods

### Materials

NADPH, NADH, dithionite, and horse heart cyt *c* were purchased from Sigma-Aldrich (St. Louis, MO, USA). YC-17 was extracted from *Streptomyces venezuelans* ATC 15439 *ΔpikC* and mevastatin sodium was prepared by saponification with 0.1 M NaOH in 96% ethanol-water at 50 °C^38,39^. The obtained compounds were verified by LC-MS. All antibiotics were obtained from SolarBio (Beijing, China). DNA polymerase restriction endonucleases were purchased from TaKaRa (Dalian, China). ClonExpress II One Step Cloning Kit were bought from Vazyme (Nanjing, China). The kits for plasmid extraction and DNA purification were purchased from Omega Bio-Tek (Jinan, China). Mutants of Fdxs were synthesized by BGI Genomics Co., Ltd. and the corresponding mutation sites were shown in Table 2. His-tagged protein purification was performed by using Ni-NTA resin (Sangon Biotech, Shanghai, China). Amicon Ultra centrifugal filters and PD-10 desalting columns were obtained from Millipore (Billerica, MA, USA) and GE Healthcare (Piscataway, NJ, USA), respectively. Oligonucleotides synthesis and DNA sequencing were conducted by Sangon Biotech (Shanghai, China).

### General experimental procedures

High-resolution Q-TOF mass spectrometry analysis was carried out with a maXis ultrahigh-resolution TOF system (Bruker Daltonik, Germany). The reaction samples were analyzed by HPLC/HRMS with a Triart C18 column (YMC Co., Ltd., Japan). Mevastatin, pravastatin, YC-17, and their derivatives were monitored at 230 nm using a linear mobile phase gradient ranging from 20% (v/v) acetonitrile to 70% (v/v) acetonitrile in 0.1% (v/v) TFA aqueous solution over 30 min. CsA and CsA-4-OH were monitored at 210 nm and analyzed in a two-buffer gradient system consisting of 25% methanol (buffer A) and 100% acetonitrile (buffer B). The gradient elution profile was as follows: 0–4 min, 40% solvent B; 4–15 min, 40–61% solvent B; 15–32 min, 61–100% solvent B; 32–35 min, 40% solvent B. The flow rate was set at 1 mL/min, and the injection volume was 20 μL.

### Protein expression and purification

The plasmids harboring certain enzyme-encoding genes used in this study are listed in Supplementary Table 2. The *adx* and *adR* genes were PCR-amplified with pKKHC-Adx_4-108_ and pBAR-bAdR as templates^40,41^ and the corresponding primers are listed in Supplementary Table 3. The truncated form of Adx (4–108 amino acids) and AdR were expressed in pCwori and pET30a with a *C*-terminal His-tag to help streamline purification. The Adx(4–108) allowed us to purify a more stable and active version of Adx^42^. All the P450s and RPs were expressed in *E. coli* (DE3) and purified via His-tag^43^. Typically, the cultures of recombinant cells in LB medium (50 mg L^−1^ Kanamycin and the rare salt solution) were grown at 37 °C (220 rpm) until OD_600_ reached 0.6–1.0 and then induced with 0.4 mM *β*-D-1-thiogalactopyranoside (IPTG) and 0.5 mM *δ*-aminolevulinic acid (ALA). The cells were cultured at 20 °C for another 20 h and centrifuged at 6000 × *g* for 10 min to pellet cells. The following protein purification was carried out using Ni-NTA affinity chromatography^38^. Purified proteins were flash-frozen by liquid nitrogen and stored at −80 °C for later use. P450 concentration was determined from CO-bound difference spectra (*ε*_450-490nm_ = 91,000 M^−1^ cm^−1^). The concentrations of redoxins and redoxin reductases were determined by measuring the absorbance at the selected wavelengths. The extinction coefficients used for calculation of concentrations were *ε*_414_ = 11,000 M^−1^ cm^−1^ for Adx^29^, *ε*_450_ = 11,300 M^−1^ cm^−1^ for AdR^44^, *ε*_415_ = 11,100 M^−1^ cm^−1^ and *ε*_455_ = 10,400 M^−1^ cm^−1^ for Pdx, *ε*_378_ = 9,700 M^−1^ cm^−1^ and *ε*_454_ = 10,000 M^−1^ cm^−1^ for PdR^25,45^.

### Extinction coefficient determination

The extinction coefficient of FAD-containing ferredoxin reductase was determined by quantitating the FAD released from the protein following guanidinium chloride treatment. The concentration of FAD was calculated at 450 nm using the extinction coefficient of 11,800 M^−1^ cm^−1^ ^46^_._ PdR was used as a control to validate the method. The ferredoxin extinction coefficient was calculated by denaturing the ferredoxin with 0.3 volumes of 12 M HCl and heating at 100 °C for 15 min. Precipitation was removed and the iron concentration was quantited at 535 nm using the extinction coefficient of 22,140 M^−1^ cm^−1^ against a blank without iron. The iron content was calculated from a (NH_4_)_2_Fe(SO_4_)_2_•6H_2_O standard curve^47^. Pdx was used as a control to validate the method.

### Ferredoxin reductase activity assay

Some flavoenzymes have the ability to reduce artificial electron acceptors with NAD(P)H (diaphorase activity). The redox compound 2,6-dichlorophenol-indophenol (DCIP) can replace Fdx as an electron acceptor in vitro. Ferredoxin reductase activity was assayed by monitoring the reduction of DCIP as the decrease of absorbance at 600 nm (*ε*_600_ = 21,800 M^−1^ cm^−1^) for 1 min^48^. One unit of ferredoxin reductase activity was defined as the amount of enzyme which required to reduce 1 μM of DCIP per sec at 30 °C. Electron donor preference was also investigated by using NADH to replace NADPH for the initiation of reactions.

### Cyt *c* reduction assays

The electron transfer efficiency of RPs was measured by monitoring the increase of reduced cyt *c* at 550 nm using extinction coefficient of 21,000 M^−1^ cm^−1^ ^49^ in a UV-visible spectrophotometer (Varian, UK). The reaction mixture containing 30 μM cyt *c*, 1 nM FdR, and varying concentrations of ferredoxin (0–40 μM) in 50 mM potassium phosphate buffer (pH 7.4)^28^. The reactions were started by adding 500 μM NAD(P)H. Steady‑state kinetic analyses were performed using OriginPro 8.5 program.

### NAD(P)H coupling efficiency

NAD(P)H coupling efficiencies of P450 reactions were determined by following the previously established procedure^24,50^. Briefly, NAD(P)H consumption was monitored at 340 nm with a SpectraMax plate reader (Molecular Devices), and calculated with the extinction coefficient of 6.22 mM^−1^ cm^−1^^ 24,51^. The substrate consumption was measured by HPLC when NAD(P)H was depleted. All measurements were performed in triplicate. The coupling efficiency was calculated as the percentage of NAD(P)H used for product formation over the total NAD(P)H consumption.

### P450 enzymatic assay

Three bacterial P450 enzymes were chosen to analyze the reactivity profile of the three Fdxs and three FdRs. The standard assay contained 1 μM P450, 10 μM Fdx, 5 μM FdR, 100 μM substrate, 0.5 mM NAD(P)^+^, and NAD(P)H regeneration system (10 mM glucose and 2 U glucose-6-phosphate dehydrogenase) in a final volume of 100 μL reaction buffer (50 mM potassium phosphate buffer, pH 7.4). The ratio of 1:10:5 for P450/Fdx/FdR was used to ensure the adequate electron supplies and hence the efficient P450-mediated conversions. CsA is poorly soluble in water. To improve the solubility and conversion rate, 10% methanol was added to the CYP-sb21 enzymatic assay mixtures containing an alternatively optimized P450/Fdx/FdR content, namely, 2 μM CYP-sb21, 20 μM Fdx, and 10 μM FdR. The reactions were incubated at 30 °C for 2 h (YC-17 and mevastatin) or 12 h (CsA) and an equal volume of MeOH was added to quench reactions and precipitate proteins. After high-speed centrifugation, the supernatants were used for HPLC and HRMS analysis.

#### Simulation of three-dimensional structure

The three-dimensional structure of P450sca-2, *Sel*FdR0978, and *Sel*Fdx1499 were modeled using AlphaFold2. Simulation of the Fdx-P450 and FdR-Fdx complex structures used the ZDOCK SERVER (ZDOCK 3.0.2). Figures 4–6 that show protein structures were prepared using PYMOL (http://www.pymol.org).

#### Statistics and reproducibility

All enzyme catalytic assays were carried out in triplicate. Statistical significance was determined by a one-factor analysis of variance (ANOVA)^52^.

### Reporting summary

Further information on research design is available in the Nature Research Reporting Summary linked to this article.

## Supplementary information

Supplementary Material

Reporting Summary

## Data Availability

The cystal structural information for proteins are available from the Protein Data Bank under the associated PDB code described in this paper. All data needed to evaluate the conclusions in the paper are present in the paper. Additional data related to this paper may be requested from the corresponding author.

## References

[1] Guengerich FP (2018). Mechanisms of cytochrome P450-catalyzed oxidations. ACS Catal..

[2] Chauhan D, Hideshima T, Anderson KC (2005). Proteasome inhibition in multiple myeloma: therapeutic implication. Annu. Rev. Pharmacol. Toxicol..

[3] Zhang X, Li S (2017). Expansion of chemical space for natural products by uncommon P450 reactions. Nat. Prod. Rep..

[4] Denisov IG, Makris TM, Sligar SG, Schlichting I (2005). Structure and chemistry of cytochrome P450. Chem. Rev..

[5] Bernhardt R, Urlacher VB (2014). Cytochromes P450 as promising catalysts for biotechnological application: chances and limitations. Appl. Microbiol. Biotechnol..

[6] Hannemann F, Bichet A, Ewen KM, Bernhardt R (2007). Cytochrome P450 systems-biological variations of electron transport chains. Biochim. Biophys. Acta.

[7] Zhang W (2014). New reactions and products resulting from alternative interactions between the P450 enzyme and redox partners. J. Am. Chem. Soc..

[8] Li Z (2020). Engineering cytochrome P450 enzyme systems for biomedical and biotechnological applications. J. Biol. Chem..

[9] Grinberg AV (2000). Adrenodoxin: structure, stability, and electron transfer properties. Proteins.

[10] Gunsalus IC (1968). A soluble methylene hydroxylase system: structure and role of cytochrome P-450 and iron-sulfur protein components. Hoppe-Seyler’s Z. Physiol. Chem..

[11] Hannemann F, Virus C, Bernhardt R (2006). Design of an *Escherichia coli* system for whole cell mediated steroid synthesis and molecular evolution of steroid hydroxylases. J. Biotechnol..

[12] Jozwik IK (2016). Structural basis of steroid binding and oxidation by the cytochrome P450 CYP109E1 from *Bacillus megaterium*. FEBS J..

[13] Zhang W (2018). Mechanistic insights into interactions between bacterial class I P450 enzymes and redox partners. ACS Catal..

[14] Cheng B, Guo H, Wang H, Zhao Q, Liu W (2021). Dissection of the enzymatic process for forming a central imidazopiperidine heterocycle in the biosynthesis of a series *c* thiopeptide antibiotic. J. Am. Chem. Soc..

[15] Ma L (2015). Reconstitution of the in vitro activity of the cyclosporine-specific P450 hydroxylase from *Sebekia benihana* and development of a heterologous whole-cell biotransformation system. Appl. Environ. Microbiol..

[16] Sun Y (2017). In vitro reconstitution of the cyclosporine specific P450 hydroxylases using heterologous redox partner proteins. J. Ind. Microbiol. Biotechnol..

[17] Li S, Ouellet H, Sherman DH, Podust LM (2009). Analysis of transient and catalytic desosamine-binding pockets in cytochrome P450 PikC from* Streptomyces venezuelae*. J. Biol. Chem..

[18] Serizawa N, Matsuoka T (1991). A two component-type cytochrome P-450 monooxygenase system in a prokaryote that catalyzes hydroxylation of ML-236B to pravastatin, a tissue-selective inhibitor of 3-hydroxy-3-methylglutaryl coenzyme A reductase. Biochim. Biophys. Acta.

[19] Ba L, Li P, Zhang H, Duan Y, Lin Z (2013). Engineering of a hybrid biotransformation system for cytochrome P450sca-2 in *Escherichia coli*. Biotechnol. J..

[20] Girhard M, Klaus T, Khatri Y, Bernhardt R, Urlacher VB (2010). Characterization of the versatile monooxygenase CYP109B1 from *Bacillus subtilis*. Appl. Microbiol. Biot..

[21] Schiffer L (2016). Metabolism of oral turinabol by human steroid hormone-synthesizing cytochrome P450 enzymes. Drug Metab. Dispos..

[22] Prasad B, Rojubally A, Plettner E (2011). Identification of camphor oxidation and reduction products in *Pseudomonas putida*: new activity of the cytochrome P450cam system. J. Chem. Ecol..

[23] Khatri Y, Schifrin A, Bernhardt R (2017). Investigating the effect of available redox protein ratios for the conversion of a steroid by a myxobacterial CYP260A1. FEBS Lett..

[24] Ba L, Li P, Zhang H, Duan Y, Lin Z (2013). Semi-rational engineering of cytochrome P450sca-2 in a hybrid system for enhanced catalytic activity: insights into the important role of electron transfer. Biotechnol. Bioeng..

[25] Sevrioukova IF, Poulos TL (2002). Putidaredoxin reductase, a new function for an old protein. J. Biol. Chem..

[26] Lambeth JD, Seybert DW, Kamin H (1979). Ionic effects on adrenal steroidogenic electron transport. The role of adrenodoxin as an electron shuttle. J. Biol. Chem..

[27] Aliverti A (1998). Probing the function of the invariant glutamyl residue 312 in spinach ferredoxin-NADP^+^ reductase. J. Biol. Chem..

[28] Xu F (2009). Crystal structure of a ferredoxin reductase for the CYP199A2 system from *Rhodopseudomonas palustris*. Proteins.

[29] Lambeth JD, Kamin H (1977). Adrenodoxin reductase and adrenodoxin. Mechanisms of reduction of ferricyanide and cytochrome *c*. J. Biol. Chem..

[30] Cramer P (2021). AlphaFold2 and the future of structural biology. Nat. Struct. Mol. Biol..

[31] Pierce BG (2014). ZDOCK server: interactive docking prediction of protein-protein complexes and symmetric multimers. Bioinformatics.

[32] Muller JJ, Lapko A, Bourenkov G, Ruckpaul K, Heinemann U (2001). Adrenodoxin reductase-adrenodoxin complex structure suggests electron transfer path in steroid biosynthesis. J. Biol. Chem..

[33] Sevrioukova IF, Poulos TL, Churbanova IY (2010). Crystal structure of the putidaredoxin reductase x putidaredoxin electron transfer complex. J. Biol. Chem..

[34] Jacquot G, Maidou-Peindara P, Benichou S (2010). Molecular and functional basis for the scaffolding role of the p50/dynamitin subunit of the microtubule-associated dynactin complex. J. Biol. Chem..

[35] Hollingsworth SA, Batabyal D, Nguyen BD, Poulos TL (2016). Conformational selectivity in cytochrome P450 redox partner interactions. Proc. Natl Acad. Sci. USA.

[36] Liang J (2020). Heterologous redox partners supporting the efficient catalysis of epothilone B biosynthesis by EpoK in *Schlegelella brevitalea*. Microb. Cell Fact..

[37] Julien B (2000). Isolation and characterization of the epothilone biosynthetic gene cluster from *Sorangium cellulosum*. Gene.

[38] Xue Y, Wilson D, Zhao L, Liu H, Sherman DH (1998). Hydroxylation of macrolactones YC-17 and narbomycin is mediated by the *pikC*-encoded cytochrome P450 in *Streptomyces venezuelae*. Chem. Biol..

[39] Yao Q (2017). Hydroxylation of compactin (ML-236B) by CYP105D7 (SAV_7469) from *Streptomyces avermitilis*. J. Microbiol. Biotechnol..

[40] Uhlmann H, Beckert V, Schwarz D, Bernhardt R (1992). Expression of bovine adrenodoxin in *E. coli* and site-directed mutagenesis of /2 Fe-2S/cluster ligands. Biochem. Biophys. Res. Commun..

[41] Sagara Y (1993). Direct expression of adrenodoxin reductase in *Escherichia coli* and the functional characterization. Biol. Pharm. Bull..

[42] Uhlmann H, Kraft R, Bernhardt R (1994). C-terminal region of adrenodoxin affects its structural integrity and determines differences in its electron transfer function to cytochrome P-450. J. Biol. Chem..

[43] Li S, Podust LM, Sherman DH (2007). Engineering and analysis of a self-sufficient biosynthetic cytochrome P450 PikC fused to the RhFRED reductase domain. J. Am. Chem. Soc..

[44] Vonrhein C (1999). Chaperone-assisted expression of authentic bovine adrenodoxin reductase in *Escherichia coli*. FEBS Lett..

[45] Holden M, Mayhew M, Bunk D, Roitberg A, Vilker V (1997). Probing the interactions of putidaredoxin with redox partners in camphor p450 5-monooxygenase by mutagenesis of surface residues. J. Biol. Chem..

[46] Prongay AJ, Engelke DR, Williams CH (1989). Characterization of two active site mutations of thioredoxin reductase from *Escherichia coli*. J. Biol. Chem..

[47] Bell SG, Dale A, Rees NH, Wong LL (2010). A cytochrome P450 class I electron transfer system from *Novosphingobium aromaticivorans*. Appl. Microbiol. Biot..

[48] Ceccarelli EA, Arakaki AK, Cortez N, Carrillo N (2004). Functional plasticity and catalytic efficiency in plant and bacterial ferredoxin-NADP(H) reductases. Biochim. Biophys. Acta.

[49] Li M, Guo W, Chen X (2016). A novel NADPH-dependent reductase of *Sulfobacillus acidophilus* TPY phenol hydroxylase: expression, characterization, and functional analysis. Appl. Microbiol. Biot..

[50] Peng HM (2016). Cytochrome b5 activates the 17,20-lyase activity of human cytochrome P450 17A1 by increasing the coupling of NADPH consumption to androgen production. Biochemistry.

[51] Yang W (2010). Molecular characterization of a class I P450 electron transfer system from *Novosphingobium aromaticivorans* DSM12444. J. Biol. Chem..

[52] Anders K (2017). Resolution of Students t-tests, ANOVA and analysis of variance components from intermediary data. Biochem. Med..

