# Supplementary material for: Three pairs of surrogate redox partners comparison for Class I cytochrome P450 enzyme activity reconstitution
Source: Commun Biol. 2022 Aug 6;5:791. doi: 10.1038/s42003-022-03764-4 (PMC9357085; doi:10.1038/s42003-022-03764-4)
Supplement: Supplementary file 1 — Supplementary Material [file 42003_2022_3764_MOESM1_ESM.pdf]

Supplemental Material for

Three pairs of surrogate redox partners comparison for Class I cytochrome P450 enzyme activity reconstitution

Xiaohui Liu[1†], Fengwei Li[1†], Tianjian Sun[1], Jiawei Guo[1], Xingwang Zhang[1,2], Xianliang Zheng[3], Lei Du[1], Wei Zhang[1], Li Ma[1*], Shengying Li [1,2]

[1]State Key Laboratory of Microbial Technology, Shandong University, Qingdao, Shandong 266237, China.

[2]Laboratory for Marine Biology and Biotechnology, Qingdao National Laboratory for Marine Science and Technology, Qingdao, Shandong 266237, China.

[3]Center For Biocatalysis and Enzyme Technology, AngelYeast Co., Ltd., Cheng Dong Avenue, Yichang, Hubei, 443003, China

*Corresponding Author E-mail: maliqd@sdu.edu.cn

[†]These authors contributed equally.

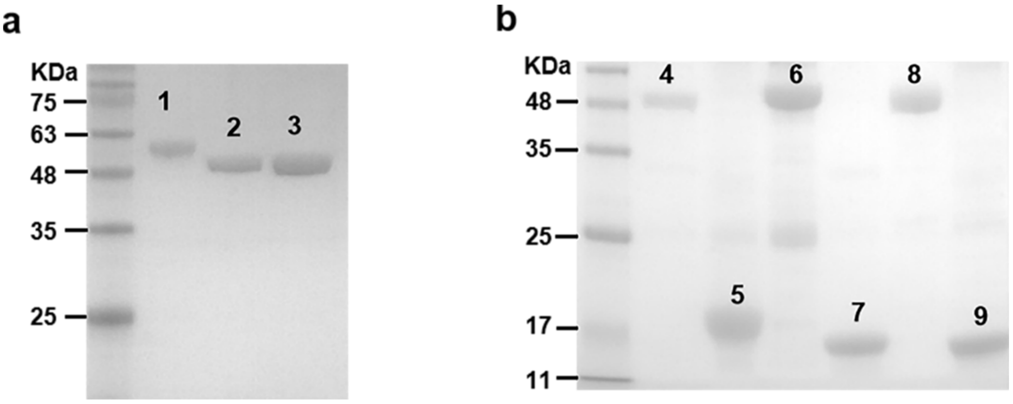

Supplementary Fig 1. SDS-PAGE analysis of purified cytochrome P450s (a) and redox partners (b). Lane 1, CYP-sb21; 2, P450sca-2; 3, PikC; 4, *Sel*FdR0978; 5, *Sel*Fdx1499; 6, AdR; 7, Adx; 8, PdR; 9, Pdx. Proteins were stained with Coomassie blue in 12.5% polyacrylamide gel.

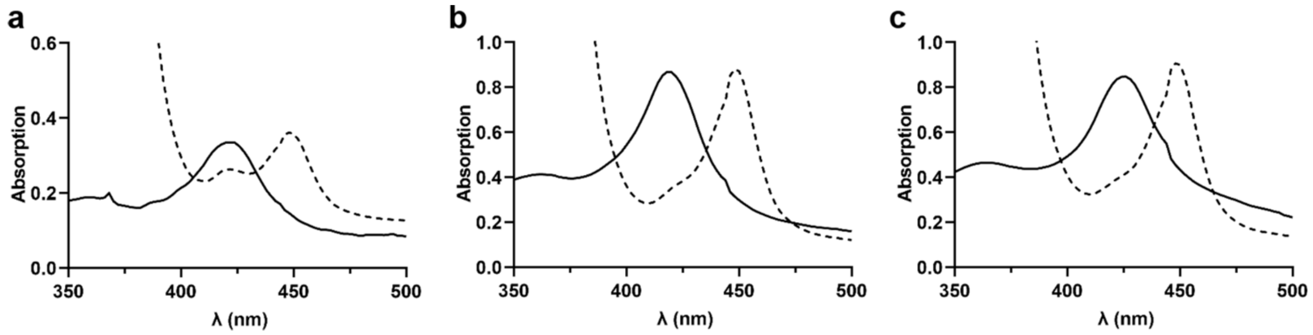

Supplementary Fig 2. CO-bound reduced difference spectra of purified P450s. (a) PikC, (b) P450sca-2, (c) CYP-sb21. *Dotted line*, P450 absorbance spectra in ferrous CO-complexed state; *Solid line*, ferric state. This assay was also employed to determine the concentrations of functional cytochrome P450 enzymes using the extinction of 91,000 $M^{-1}cm^{-1}$ [1,2].

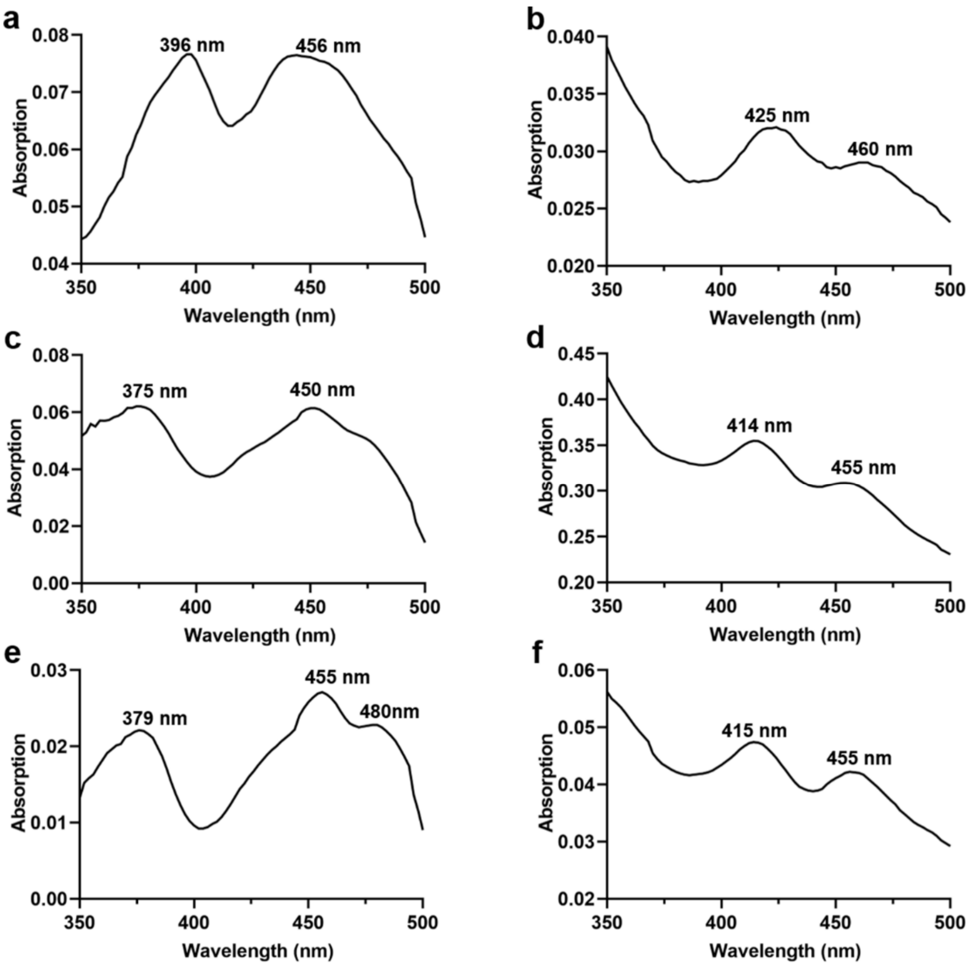

Supplementary Fig 3. UV/Vis absorption spectra of the selected redox partner proteins. (a) *Sel*FdR0978, (b) *Sel*Fdx1499, (c) AdR, (d) Adx, (e) PdR, and (f) Pdx.

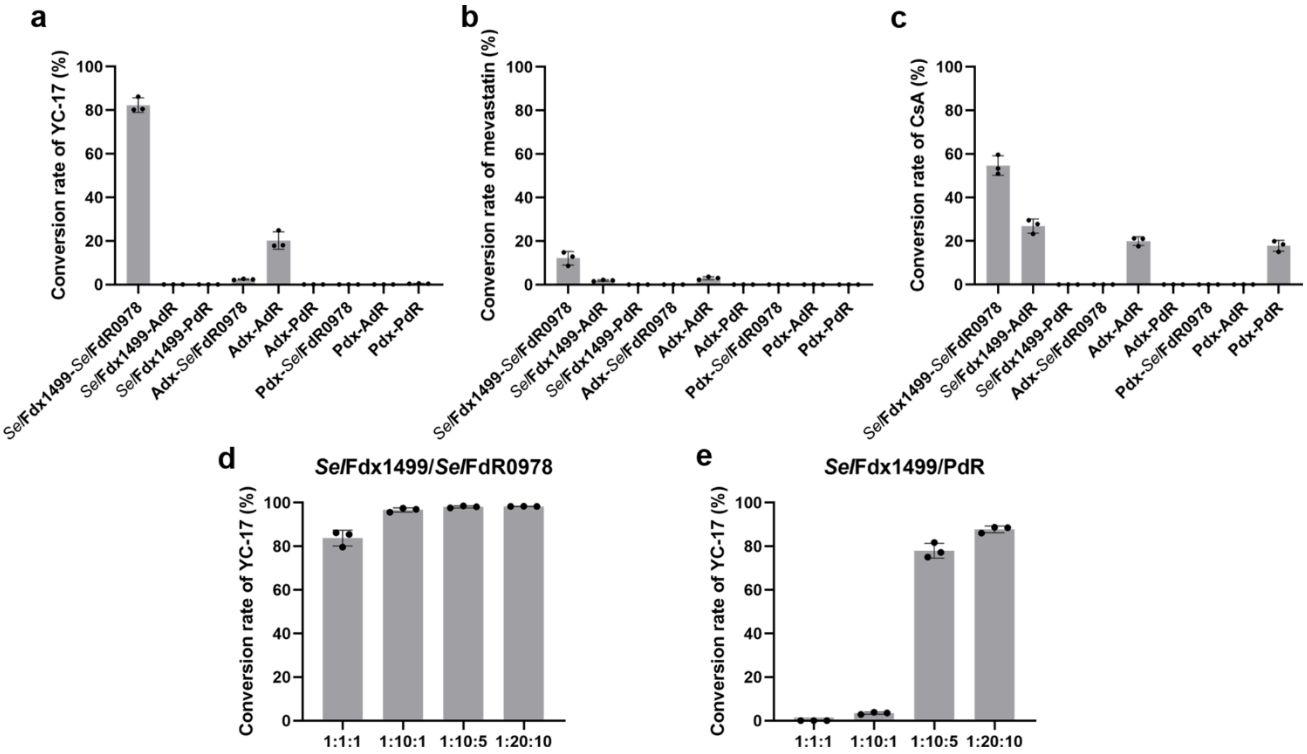

Supplementary Fig 4. The effects of different P450/Fdx/FdR ratios on P450-mediated catalysis. The catalytic activities of PikC (a), P450sca-2 (b), and CYP-sb21 (c) when supported by an equal molar ratio of P450, Fdx and FdR. The catalytic activities of PikC when supported by different ratios of *Sel*Fdx1499/*Sel*FdR0978 (d) and *Sel*Fdx1499/PdR (e). All the experiments were carried out in triplicate. The error line represents the standard deviation. *P* values in each groups were calculated with single factor ANOVA analysis, and all *P* values were less than 0.01.

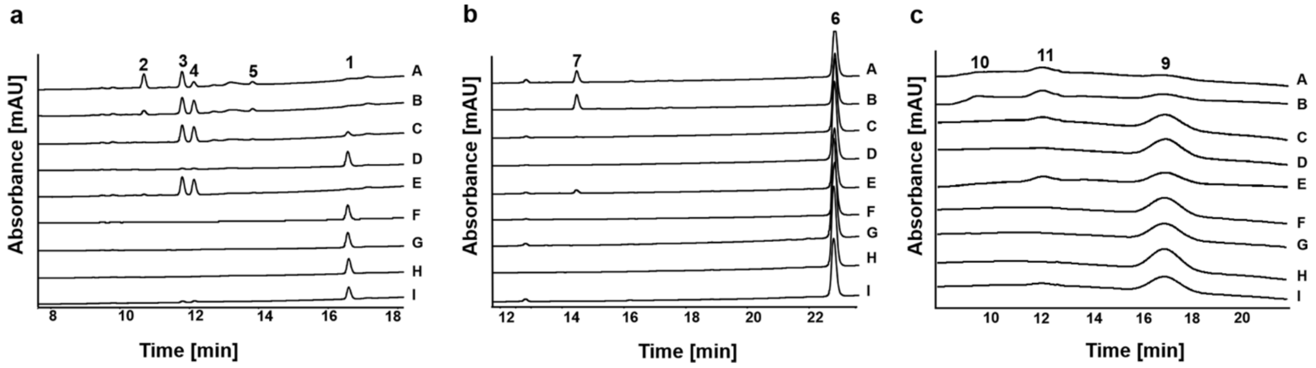

Supplementary Fig 5. HPLC analysis of three P450 enzymatic reactions. (a) PikC hydroxylates YC-17 (**1**) at either C10 or C12 position, giving rise to neomethymycin (**3**), methymycin (**4**), novamethymycin (**2**), and ketomethymycin (**5**). (b) P450sca-2 converts mevastatin (**6**) into pravastatin (**7**). (c) CYP-sb21 hydroxylates CsA (**9**) into CsA-9-OH (**10**) and/or CsA-4-OH (**11**) to different extents. A, *Sel*Fdx1499/*Sel*FdR0978; B, *Sel*Fdx1499/AdR; C, *Sel*Fdx1499/PdR; D, Adx/*Sel*FdR0978; E, Adx/AdR; F, Adx/PdR; G, Pdx/*Sel*FdR0978; H, Pdx/AdR; I, Pdx/PdR.

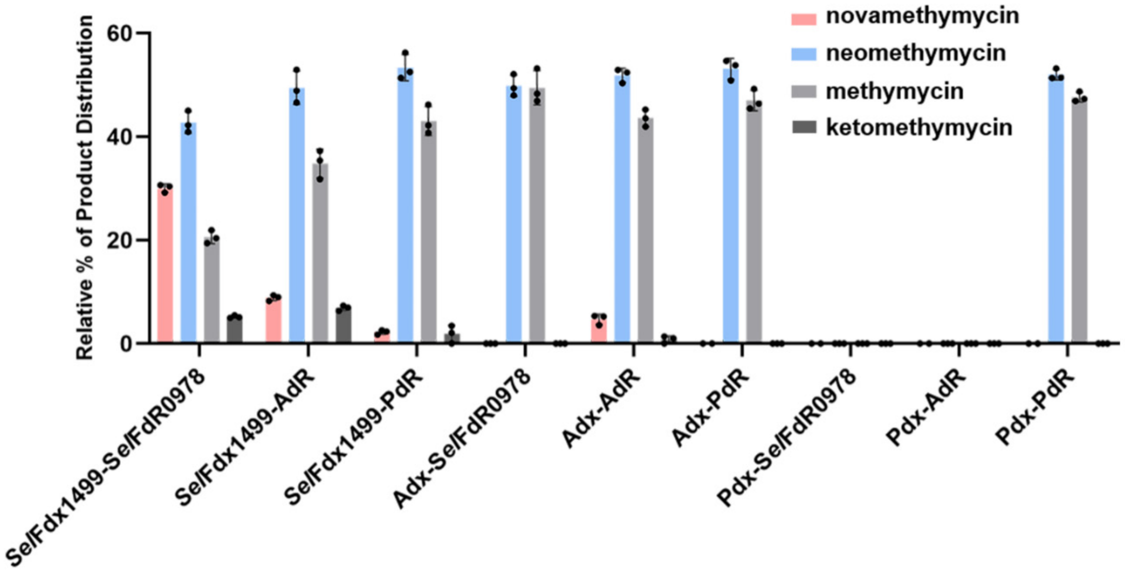

Supplementary Fig 6. Products distribution of PikC when coupled with 9 different combinations of redox partners. The diagram shows the relative percentage of products (%) obtained from the *in vitro* conversions of YC-17 by PikC in the presence of GDH/glucose as NAD(P)H recycling system. The error line represents the standard deviation. *P* values in each groups were calculated with single factor ANOVA analysis, and all *P* values were less than 0.01.

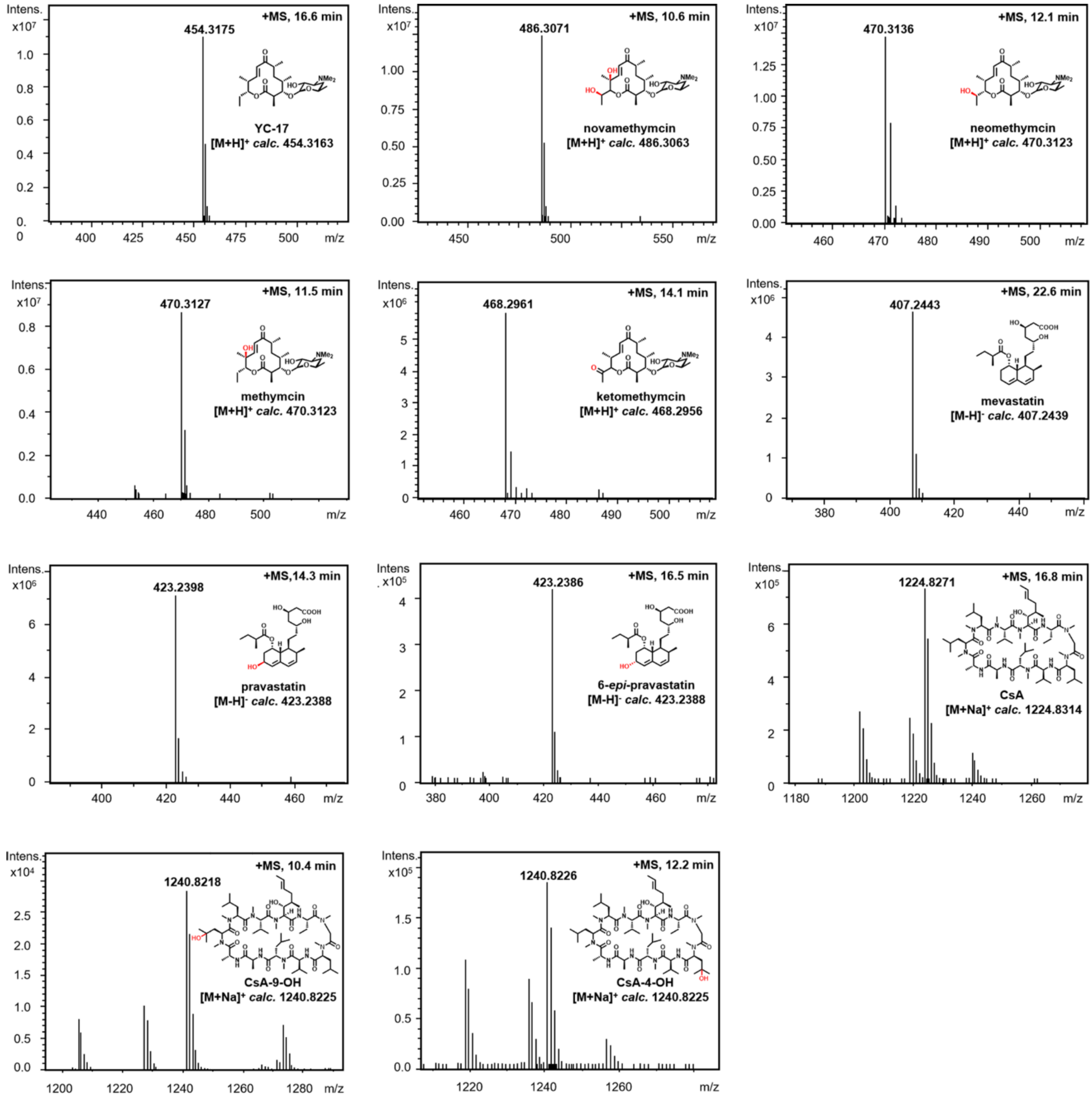

Supplementary Fig 7. High resolution mass spectra of the substrates and products associated with the three P450-mediated reactions.

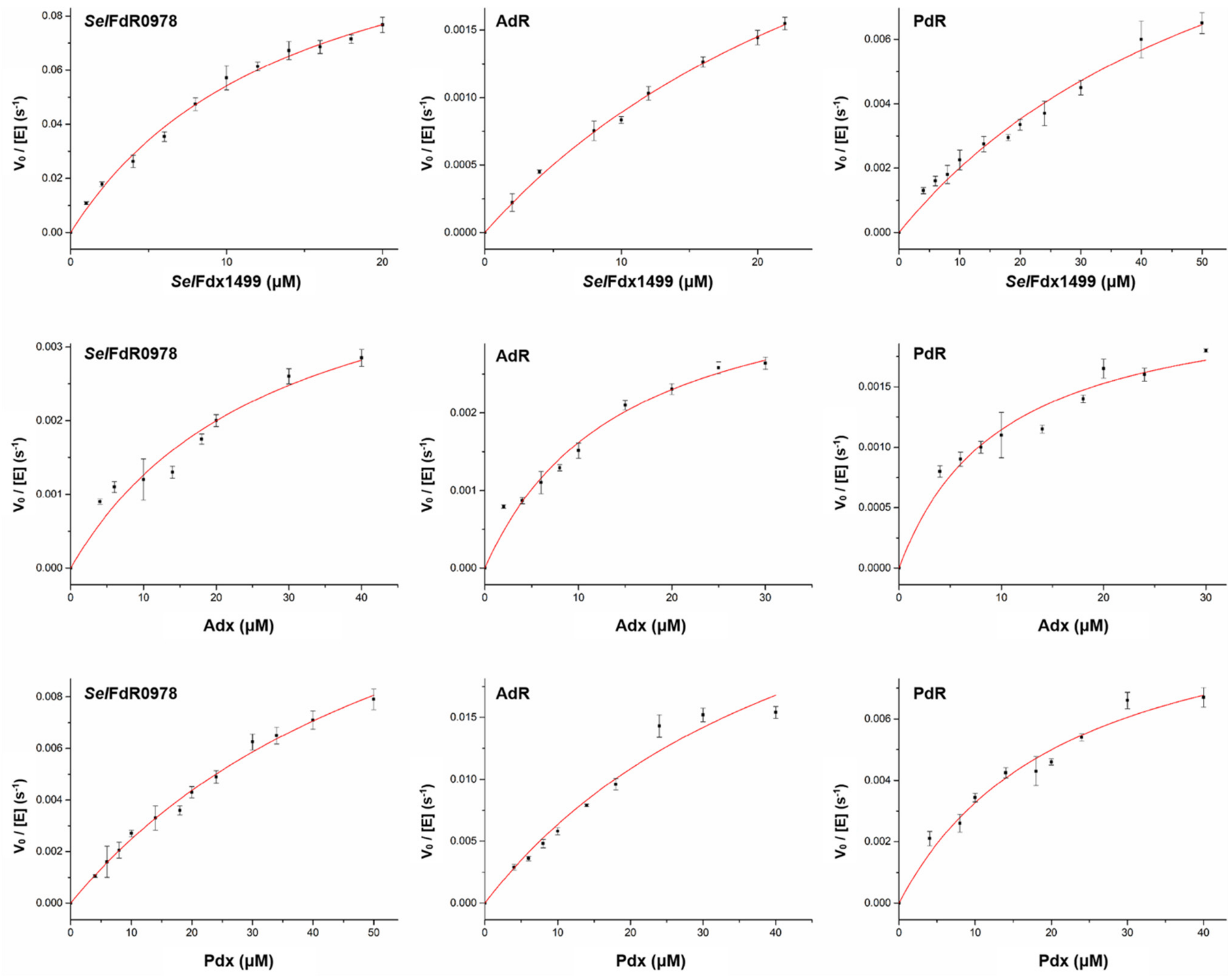

Supplementary Fig 8. The steady-state kinetic curves of cytochrome *c* (cyt *c*) reduction by 9 pairs of redox partners. In the electron transfer assays, cyt *c* was used as the final electron acceptor. The assays were carried out on a SpectraMax M2 plate reader (USA) at 30 °C by varying the concentrations of Fdxs in 50 mM sodium phosphate buffer (pH 7.4).

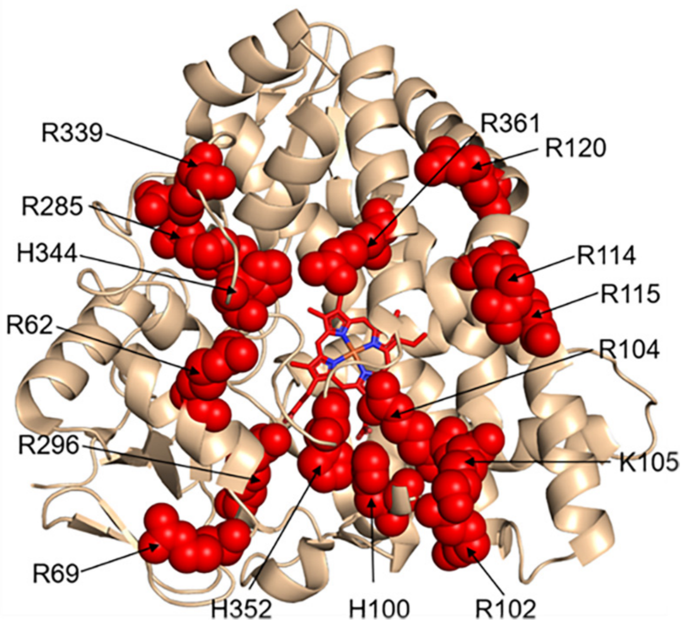

Supplementary Fig 9. The conservative basic amino acids on the proximal surface of the three P450 enzymes. Basic amino acids R, H or K in different P450s were mapped onto the proximal surface of PikC, which form a conservative positively charged surface.

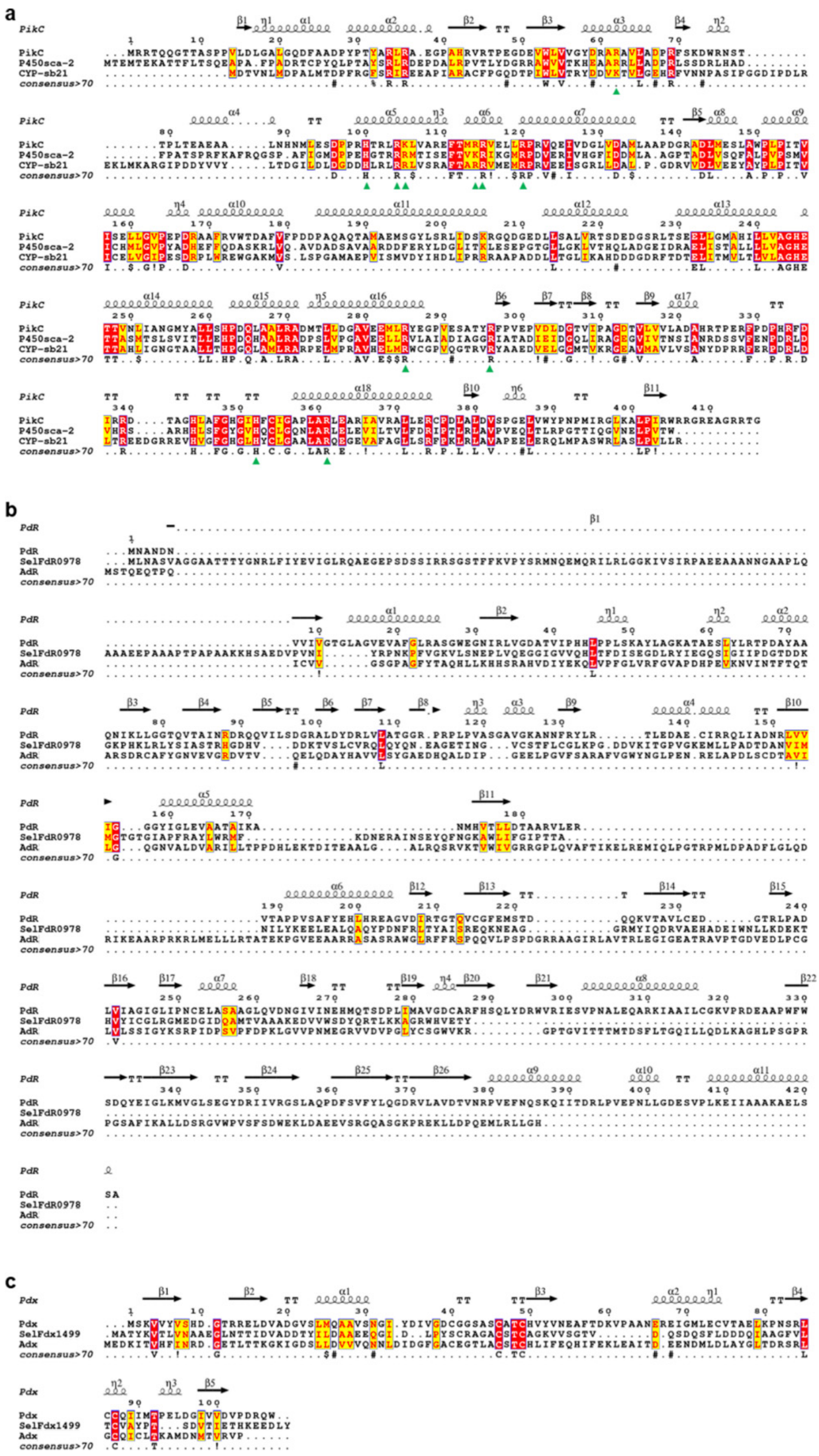

Supplementary Fig 10. Protein sequence alignment of the studied P450s (a), Fdxs (b) and FdRs (c). The secondary structure alignments are based on the models of PikC (PDB ID: 2BVJ), PdR (PDB ID: 1Q1R), and Pdx (PDB ID: 1VJI). The conservative basic amino acids R, H and K on the proximal surface of P450s are stamped by green triangles. Sequence alignment based on their tertiary structures was prepared using T-COFFEE online service [3,4] and the figure was output by ESPript 3.0 [5].

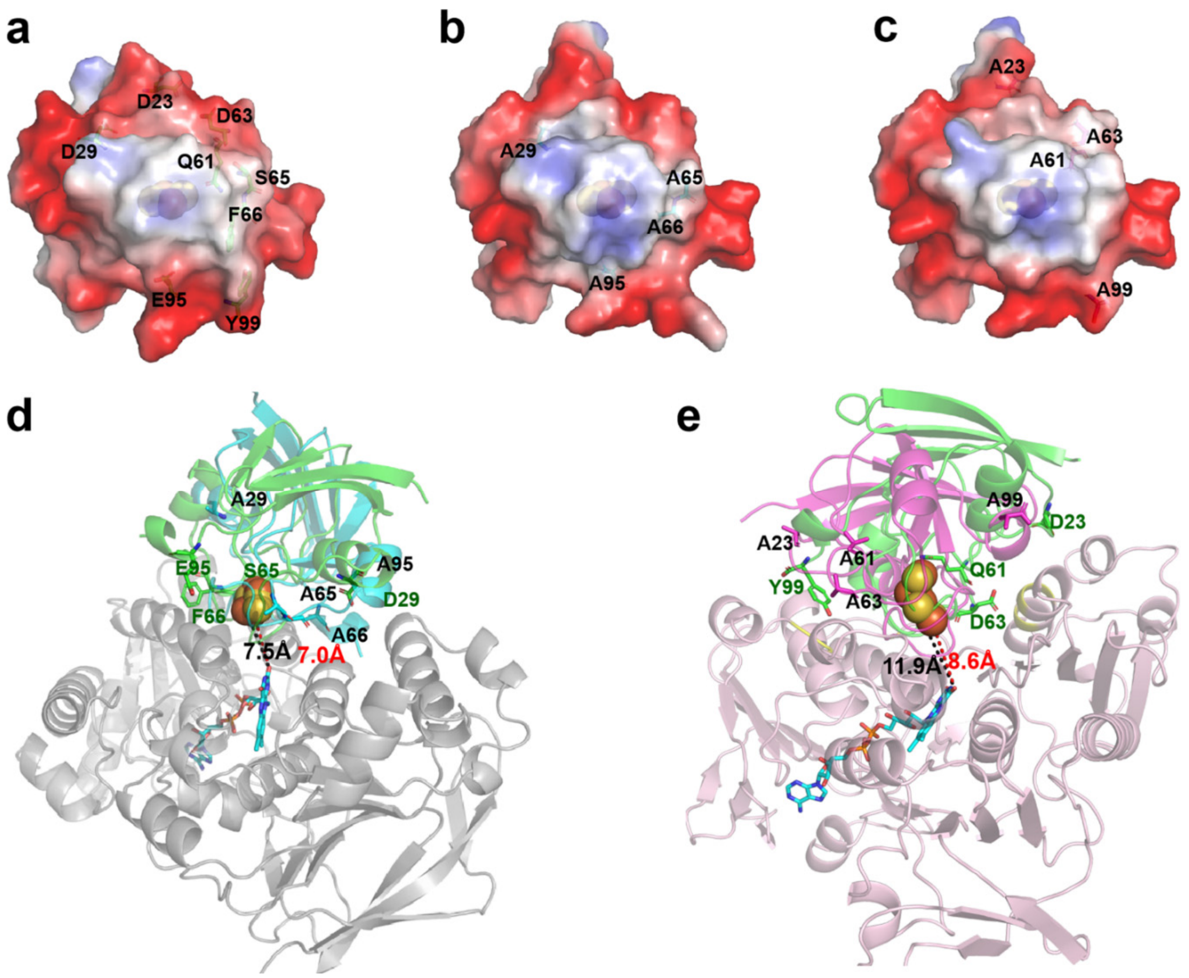

Supplementary Fig 11. The electrostatic surface and interface analysis of AdR-*Sel*Fdx1499, and PdR-*Sel*Fdx1499 before and after mutagenesis. The electrostatic surface analysis of *Sel*Fdx1499 (a), *Sel*Fdx1499-M2 (b), and *Sel*Fdx1499-M3 (c). Positively and negatively charged surfaces are colored in blue and red, respectively. Docking models of *Sel*Fdx1499 mutants in complex with AdR (d) and PdR (e). The wild-type *Sel*Fdx1499 is shown in green and the key interacting residues on AdR/PdR-*Sel*Fdx1499 interfaces are shown as sticks. When replaced by alanine, the mutant *Sel*Fdx1499 coupled with AdR or PdR is shown in cyan (d) and pink (e). FAD is shown as sticks in cyan. The Fe_2S_2 cluster is shown as spheres. The shortest FAD-Fe_2S_2 distances (Å) of wide-type and mutant-type are indicated by black and red dashed lines, respectively.

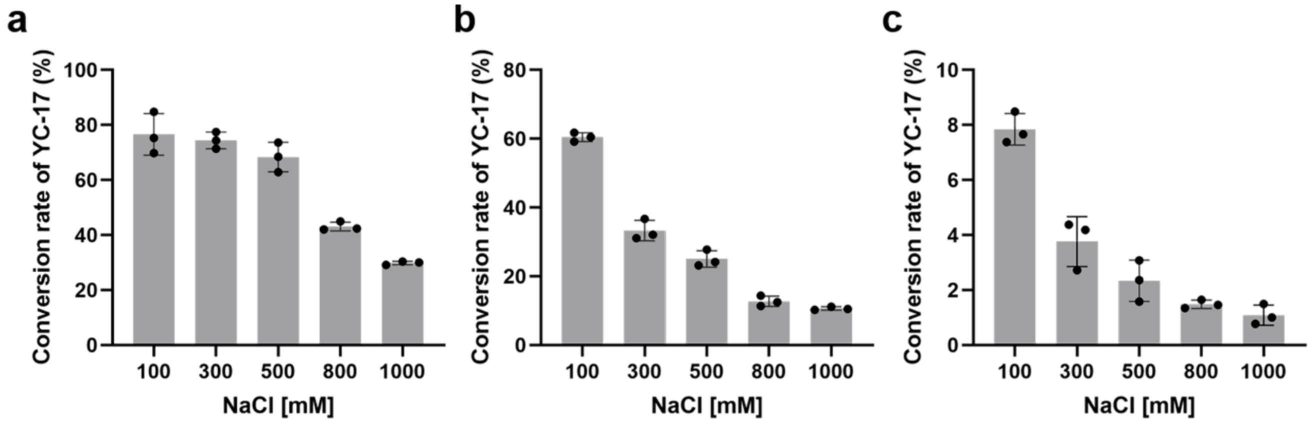

Supplementary Fig 12. Conversions of YC-17 by PikC/*Sel*Fdx1499/*Sel*FdR0978 (a), PikC/Adx/AdR (b), and PikC/Pdx/PdR (c) under different NaCl concentrations. All the experiments were carried out in triplicate. P<0.01, P-value was calculated with single factor ANOVA analysis. The error line represents the standard deviation. *P* values in each groups were calculated with single factor ANOVA analysis, and all *P* values were less than 0.01.

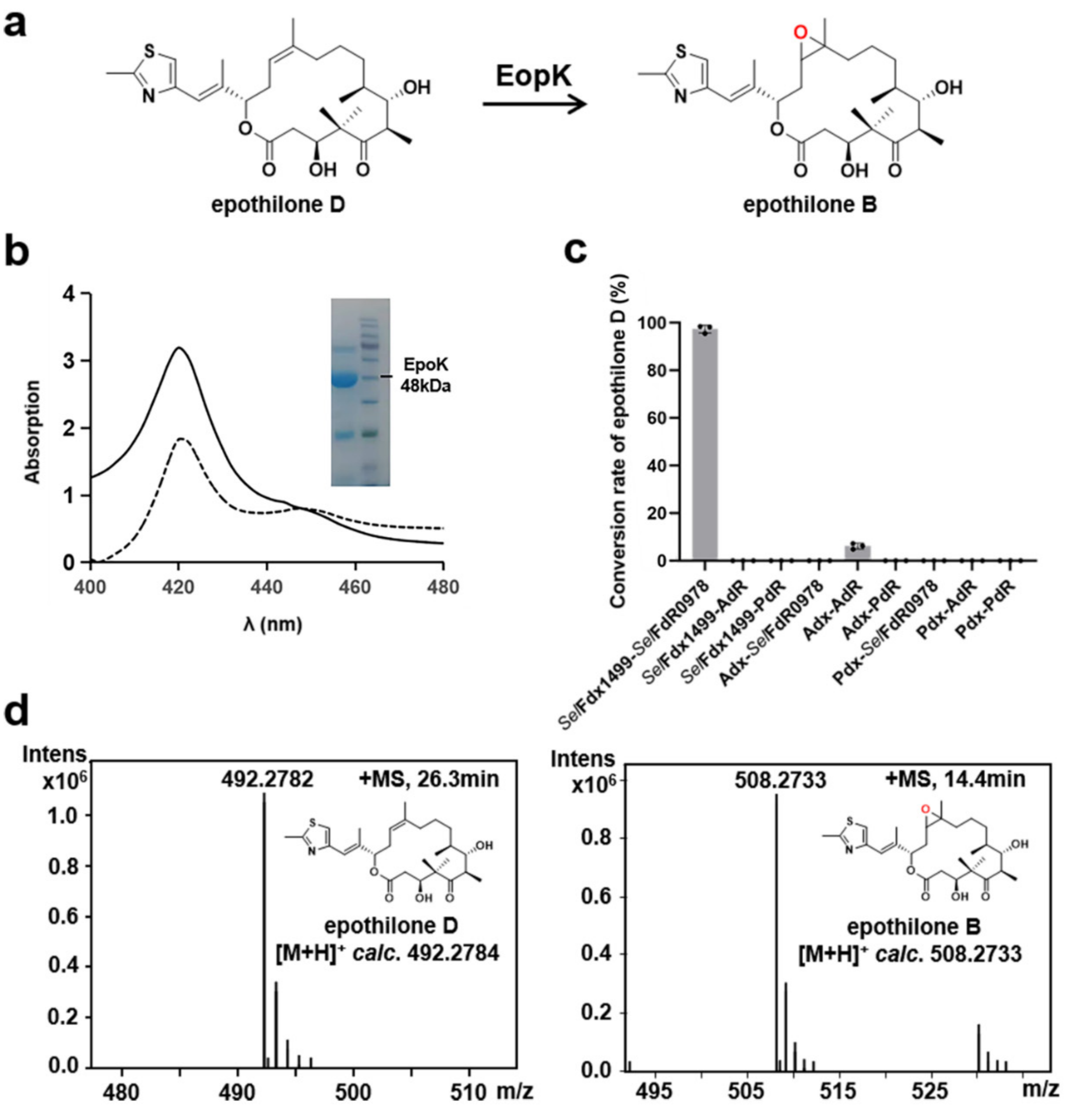

Supplementary Fig 13. Conversion of epothilone D to B catalyzed by EpoK. (a) The EpoK-catalyzed reaction. (b) The CO-bound reduced difference spectrum of purified EpoK. (c) The catalytic activities of EpoK when supported by nine different combinations of redox partners. (d) High resolution mass spectra of the substrate and product associated with the EpoK-mediated reaction.

Supplementary Table 1. Coupling efficiency for 9 redox partner pairs measured with PikC, P450sca-2 and CYP-sb21. "-" means no product was detected.

	**PikC**<br>**Coupling efficiency (%)**	**P450sca-2**<br>**Coupling efficiency (%)**	**CYP-sb21**<br>**Coupling efficiency (%)**
*Sel*Fdx1499/*Sel*FdR0978	85.6 ± 2.1	86.4 ± 5.3	12.6 ± 1.8
*Sel*Fdx1499/AdR	76.1 ± 1.8	4.8 ± 0.9	3.2 ± 0.5
*Sel*Fdx1499/PdR	9.4 ± 1.7	14.9 ± 2.2	1.2 ± 0.5
Adx/*Sel*FdR0978	16.4 ± 0.2	-	-
Adx/AdR	83.1 ± 3.2	20.1 ± 1.3	5.0 ± 2.3
Adx/PdR	9.4 ± 6.7	-	-
Pdx/*Sel*FdR0978	-	-	-
Pdx/AdR	-	-	-
Pdx/PdR	72.4 ± 4.0	-	3.3 ± 0.4

Supplementary Table 2. Strains and plasmids used in this study.

E. coli **strain or plasmid**	**Relevant characteristics**	**Reference/Source**
**Strains**		
DH5α	Cloning host	Life Technologies
BL21(DE3)	Expression host	Life Technologies
**Plasmids**		
pET28b-*pikC*	Plasmid pET28b harboring *pikC* gene	Wei Zhang et al. [6]
pET28b-*P450sca-2*	Plasmid pET28b harboring *P450sca-2* gene	Wei Zhang et al. [6]
pET28b-*cyp-sb21*	Plasmid pET28b harboring *cyp-sb21* gene	Li Ma et al. [7]
pET28b-*selfdR*0978	Plasmid pET28b harboring *selfdR*0978 gene	Li Ma et al. [7]
pET28b-*selfdx*1499	Plasmid pET28b harboring *selfdx*1499 gene	Li Ma et al. [7]
pCwori$^+$-*adR*	Plasmid pCwori$^+$ harboring *adR* (4-108) gene, added His-tag	This work
pET30a-*adx*	Plasmid pET30a harboring *adx* gene, added His-tag	This work
pET28b-*pdx*	Plasmid pET28b harboring *pdx* gene	Wei Zhang et al. [6]
pET19b-*pdR*	Plasmid pET19b harboring *pdR* gene	Wei Zhang et al. [6]
pET28b-*selfdx1499-M1-M4*	Plasmid pET28b harboring *selfdx1499-M1-M4* gene	This work
pET30a-*adx-M1-M4*	Plasmid pET30a harboring *adx-M1-M4* gene	This work
pET28b-*pdx-M1-M4*	Plasmid pET28b harboring *pdx-M1-M4* gene	This work

Supplementary Table 3. Primers used in this study

Oligonucleotide	Primer sequence (5'-3')	Function of underlined bases
Adx-F	CGGGGTACCATGGAAGATAAAATAACAGTCC	*Kpn* I restriction site
Adx-R	CCCTCGAGTTAAGGTACTCGAACAGTC	*Xho* I restriction site
AdR-F	GGAATTCCATATGAGCACTCAAGAACAAACTC	*Nde* I restriction site
AdR-R	CCCTCGAGGTGCCCCAGCAGCCGCAGCA	*Xho* I restriction site

Supplementary References

1 Li, S., Podust, L. M. & Sherman, D. H. Engineering and analysis of a self-sufficient biosynthetic cytochrome P450 PikC fused to the RhFRED reductase domain. *J. Am. Chem. Soc.* **129**, 12940-12941 (2007).

2 Omura, T. & Sato, R. The carbon monoxide-binding pigment of liver microsomes. II. solubilization, purification, and properties. *J. Biol. Chem.* **239**, 2379-2385 (1964).

3 Notredame, C., Higgins, D. G. & Heringa, J. T-Coffee: A novel method for fast and accurate multiple sequence alignment. *J. Mol. Biol.* **302**, 205-217 (2000).

4 Di Tommaso, P. *et al.* T-Coffee: a web server for the multiple sequence alignment of protein and RNA sequences using structural information and homology extension. *Nucleic Acids Res.* **39**, W13-17 (2011).

5 Robert, X. & Gouet, P. Deciphering key features in protein structures with the new ENDscript server. *Nucleic Acids Res.* **42**, W320-324 (2014).

6 Zhang, W. *et al.* Mechanistic insights into interactions between bacterial class I P450 enzymes and redox partners. *ACS Catal.* **8**, 9992-10003 (2018).

7 Ma, L. *et al.* Reconstitution of the *in vitro* activity of the cyclosporine-specific P450 hydroxylase from *Sebekia benihana* and development of a heterologous whole-cell biotransformation system. *Appl. Environ. Microbiol.* **81**, 6268-6275 (2015).
